# How do turbidite systems behave from the hydrogeological point of view? New insights and open questions coming from an interdisciplinary work in southern Italy

**DOI:** 10.1371/journal.pone.0268252

**Published:** 2022-05-06

**Authors:** Pietro Rizzo, Edoardo Severini, Antonio Bucci, Federico Bocchia, Giuseppe Palladino, Nicolò Riboni, Anna Maria Sanangelantoni, Roberto Francese, Massimo Giorgi, Paola Iacumin, Federica Bianchi, Claudio Mucchino, Giacomo Prosser, Domenico Mazzone, Dario Avagliano, Francesco Coraggio, Antonella Caputi, Fulvio Celico

**Affiliations:** 1 Department of Chemistry, Life Science and Environmental Sustainability, University of Parma, Parma, Parma, Italy; 2 Department of Biosciences and Territory, University of Molise, Pesche, Isernia, Italy; 3 Department of Sciences, University of Basilicata, Potenza, Potenza, Italy; 4 National Institute of Oceanography and Applied Geophysics–OGS, Sgonico, Trieste, Italy; 5 Proger S.p.A., San Giovanni Teatino, Chieti, Italy; 6 ENI S.p.A., Viggiano, Potenza, Italy; “G. d’Annunzio” University of Chieti and Pescara, ITALY

## Abstract

Turbidite successions can behave either as aquitards or aquifers depending on their lithological and hydraulic features. In particular, post-depositional processes can increase rock permeability due to fracture development in the competent layers. Thus, at a local scale, turbidite systems warrant further detailed investigations, aimed at reconstructing reliable hydrogeological models. The objective of this work was to investigate from the hydrogeological perspective a turbiditic aquifer located in southern Italy, where several perennial and seasonal springs were detected. Considering the complex hydrodynamics of these systems at the catchment scale, to reach an optimal characterization, a multidisciplinary approach was adopted. The conceptual framework employed microbial communities as groundwater tracers, together with the physicochemical features and isotopic signature of springs and streams from water samples. Meanwhile, geophysical investigations coupled with the geological survey provided the contextualization of the hydrogeological data into the detailed geological reconstruction of the study area. This *modus operandi* allowed us to typify several differences among the samples, allowing identification of sources and paths of surface water and groundwater, along with diffuse groundwater outflow along streams. As a final result, a hydrogeological conceptual model was reconstructed, underlining how at a very local scale the lithologic, hydraulic, and geomorphological heterogeneity of the studied relief can lead to an improved hydrogeological conceptual model compared to that of other turbidite systems. These results open new questions about the hydrogeological behavior of turbiditic aquifers, which could be pivotal in future research. In fact, these systems could support relevant ecosystems and anthropic activities, especially where climate change will force the research of new (and probably less hydrogeologically efficient) water sources.

## Introduction

Turbidite successions commonly consist of porous and permeable marine graded sandstone and conglomerate beds alternating with less permeable mudstones. Their ability to behave as aquifers or aquitards strongly depends on the geometry of the reservoir lithologies and their connectivity (e.g., [1]). For example, lens-shaped turbidites will be completely isolated in the surrounding mudstones forming only limited reservoirs. On the contrary, turbidite sheets, with erosional basal surfaces the remove the mudstone interbeds, can form thick interconnected permeable packages. Post-emplacement tectonics can also affect turbidite permeability by reducing (through occlusion of pores due to compaction, juxtaposition of permeable and impermeable strata, and clay smear) or increasing (fracturing) the porosity and connectivity (e.g., [2]).

Due to their lithological and hydraulic features, turbidites are not used as primary sources of groundwater for human purposes everywhere because they are often schematized as aquitards in hydrogeological contexts, both at regional or catchment scales (e.g., [1]). This is typical in countries like Italy, where large alluvial and/or carbonate aquifers supply enough groundwater to support drinking, rural, and industrial needs [3]. However, purpose-designed studies have demonstrated that these sedimentary sequences do not necessarily behave as aquitards [4, 5] and can be characterized by complex hydrodynamics at the catchment scale [6, 7], with significant vertical heterogeneity (from a hydraulic perspective) where stress-release fracturing and/or rock alteration enhance rock permeability in the near-surface medium [8, 9].

The permeability contrast between the more permeable sandstone and the less permeable layers can cause the groundwater to flow out at the contact between these stratigraphic layers, producing numerous springs, even though significant differences in the number and density of springs can be observed depending on the “macro-exposure” (*sensu [10]*). In detail, Mocior et al. [10] found a higher density of springs on slopes exposed opposite to the rock layer dip, but the higher-discharge springs were detected along the slopes consistent with the rock strata dip. Some authors also suggested the influence of tectonic contacts between turbidites and the underlying shales and large rock-block slides (e.g. [11]). Simultaneously, spring location can be influenced by low-permeability fault zones within the rock mass (e.g., [12]) characterized by no- or low-flow cores [13–15]. Moreover, considering the possible variation in permeability with depth [8], spring presence and location could also be associated with perched (often temporary) aquifers overlying deeper perennial groundwater, as noted in other low-permeability systems [16].

The final aim of this work is to expand the knowledge of hydrogeological behavior of turbidite sequences; this also takes into account the now inevitable climate change and the need to study sets of springs even with reduced discharge that could play an essential role in future water supplies [17, 18]. Given the geologic complexity of the investigated area, an interdisciplinary approach was preferred. In fact, this procedural design was reported as a powerful approach in the characterization of complex hydrogeological models (e.g., [16, 19, 20]), where interpretations from the individual disciplines could have led to a (possibly) less accurate understanding of the hydrogeological processes. Thus, the reported hydrogeological conceptual model was deduced by merging geological and geophysical surveys with hydrogeological, chemical, isotopic, and microbiological investigations. On one side, geological and geophysical surveys were proposed to better characterize the study area stratigraphically. On the other side, chemical, isotopic, and microbiological investigations were performed to better identify the (bio)geochemical processes affecting and shaping groundwater behaviors noted in hydrogeological investigations.

### Geological setting

**Fig 1:**
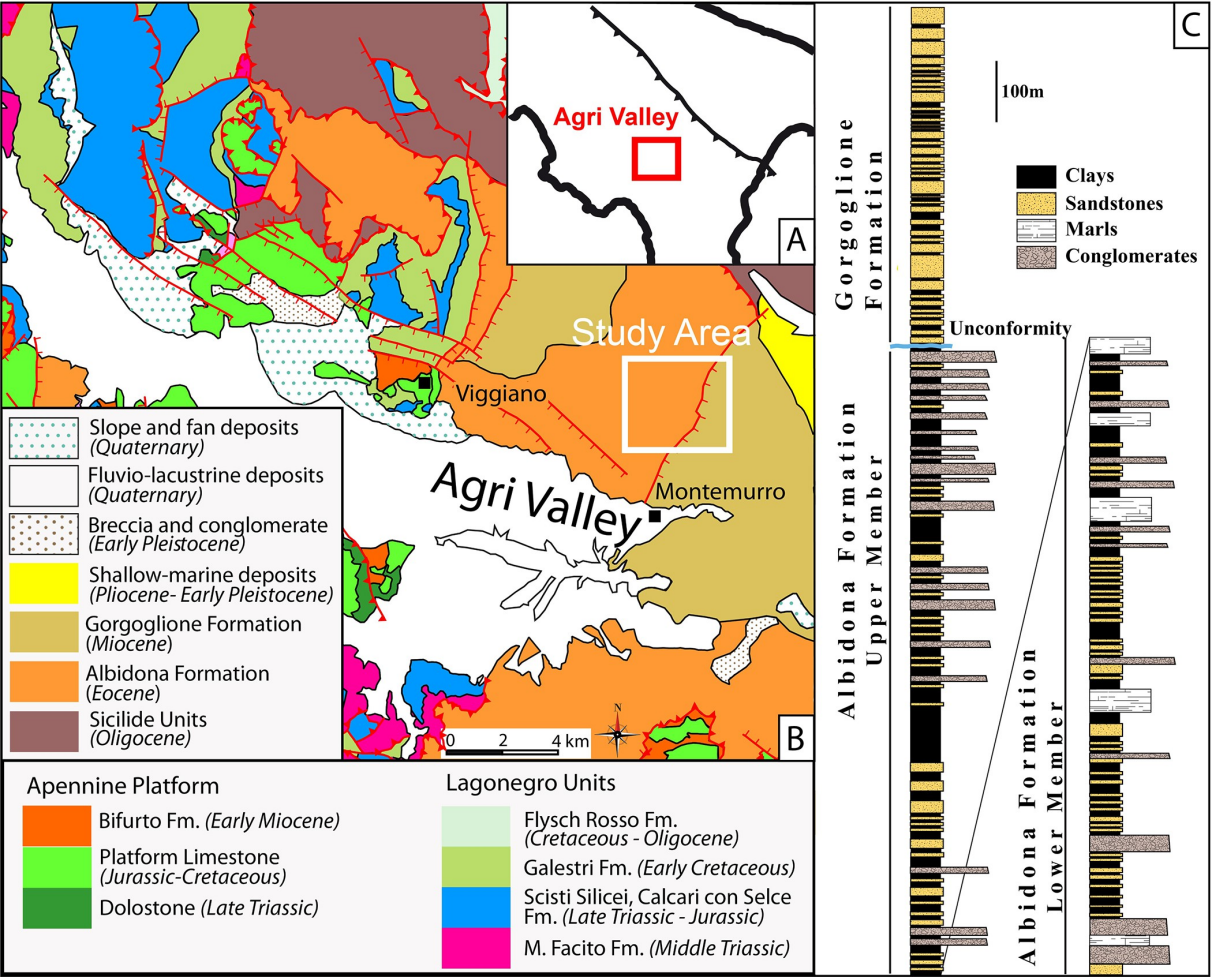
Sketch map of the Southern Apennines (A), schematic geological map of the Agri Valley (B) and stratigraphic column of the Albidona and Gorgoglione Formations (C). After Prosser et al. [26], modified.

**Fig 2:**
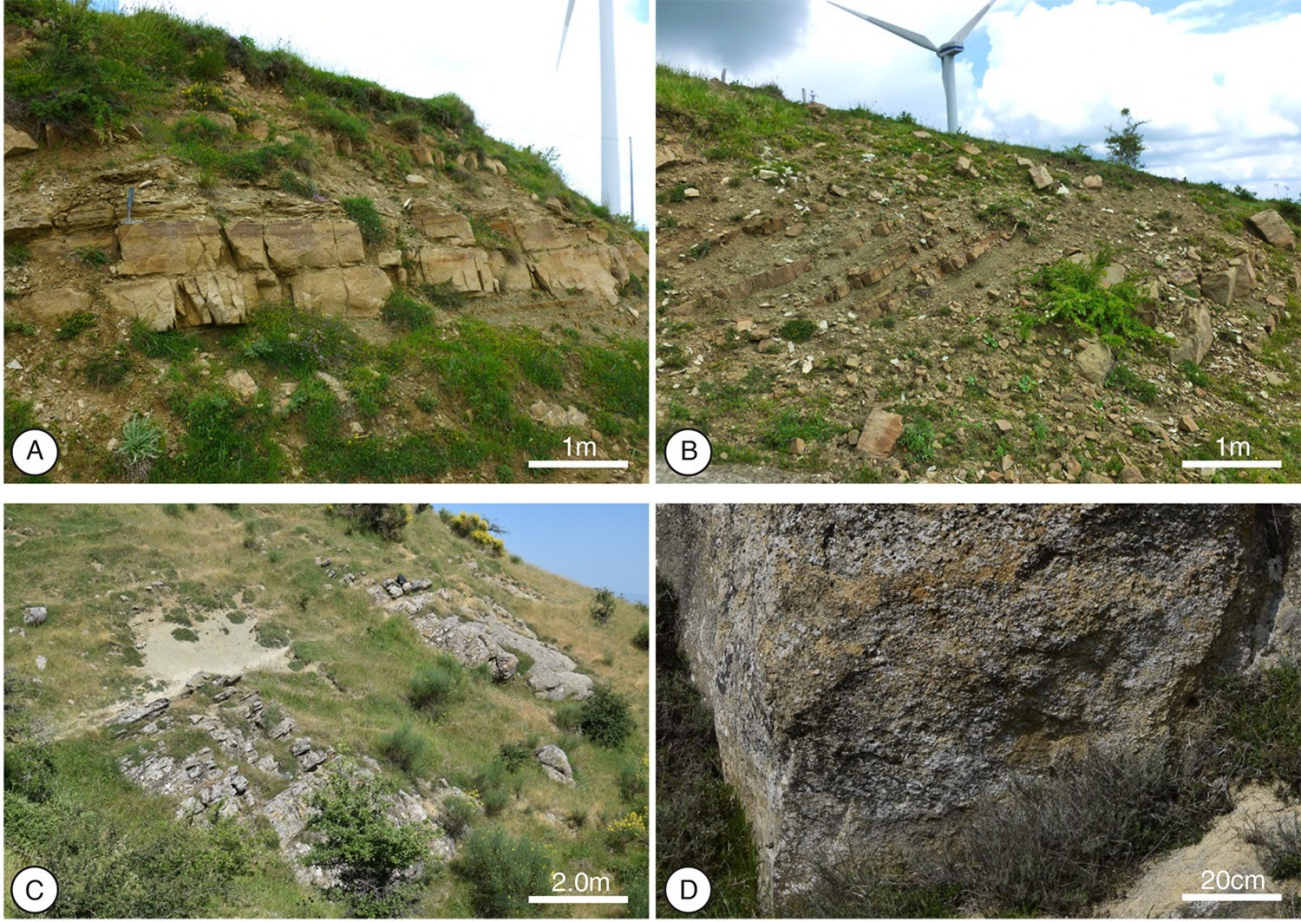
Albidona Formation outcrops recognized between Monte dell’Agresto and Contrada La Rossa. (A) Alternating, dm-thick graded turbidite sandstones and clays (AAPD); (B) Regularly alternating, thin-bedded sandstones and clays (BARD); (C) Crudely bedded marls (MRN); (D) microconglomerates (BCMD).

**Fig 3:**
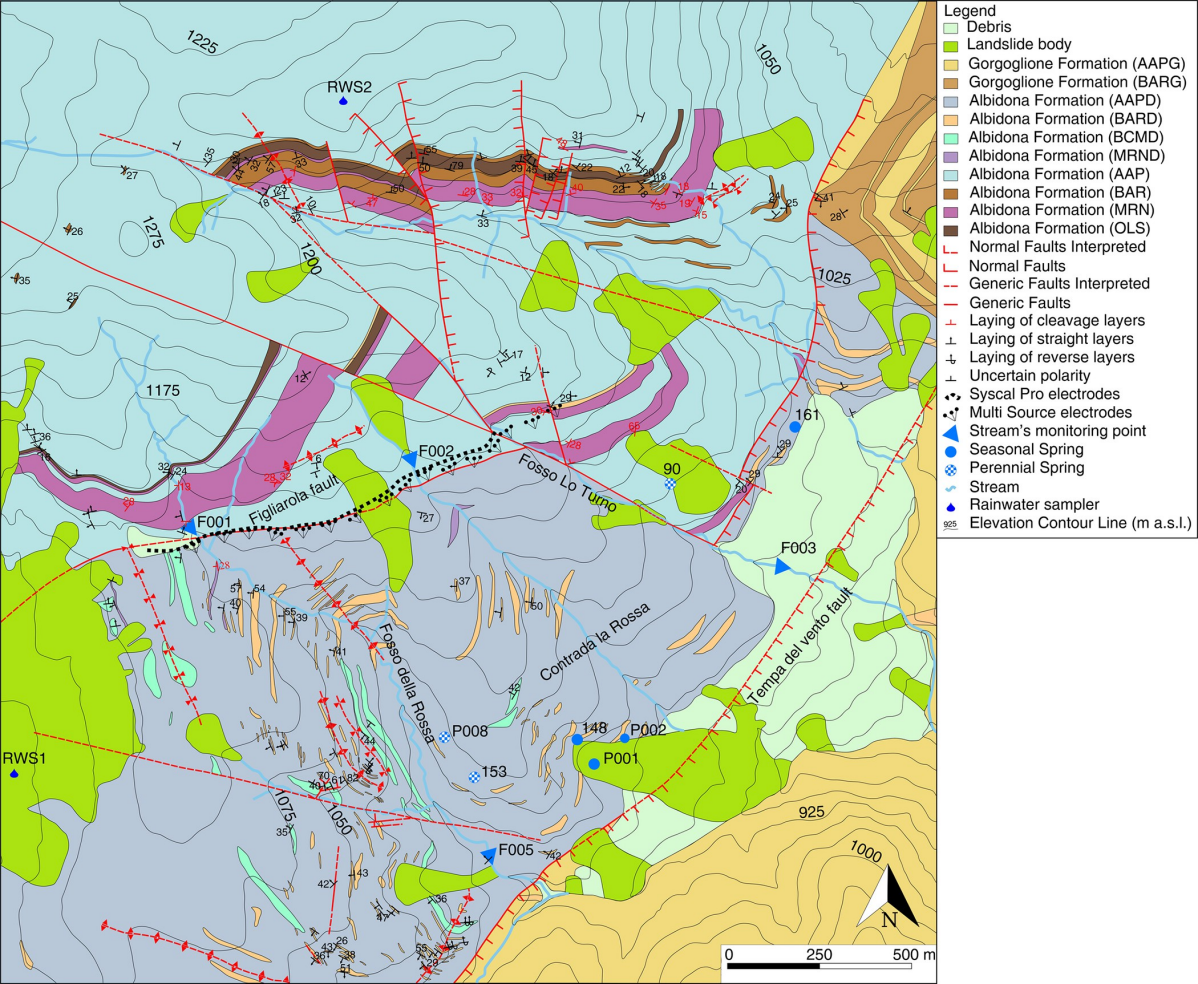
Geological map, geophysical investigation, and monitored springs/streams. After Prosser et al. [26], modified.

The studied turbidite sequences are exposed in the Montemurro area along the southeastern side of the high Agri Valley (Figs 1 and 2). This consists of a Quaternary intermontane fault-controlled basin that occupies the axial portion of the southern Apennines fold and thrust belt [21, 22] (Fig 1A and 1B). The geological setting of the study area is mainly the result of a complex tectonic history starting with Cenozoic contractional tectonics, leading to the building of the Southern Apennines fold and thrust belt, followed by strike-slip and extensional tectonics during the Quaternary [23]. The Agri valley exposes the main tectonic units that make up the Southern Apennines fold and thrust belt. The geometrically lower unit consist of deep basin sediments of Mesozoic age (Lagonegro Units; Fig 1B), divided into sandstone, limestone and radiolarite of the M. Facito Formation (Middle Triassic), limestone with chert nodules grading upwards to reddish shales and siliceous rocks (Calcari con Selce and Scisti Silicei Formations of Upper Triassic to Jurassic age), brown shales with intercalations of marly limestone beds of the Galestri Formation (Early Cretaceous) and reddish shales with thick calcarenite intercalations of the Flysch Rosso Formation (Late Cretaceous—Oligocene). The Lagonegro Units are covered along a shallow dipping thrust contact by the carbonates of the Apennine Platform Unit (Fig 1B), made up of dolostones of Early Triassic age, followed upwards by Jurassic and Cretaceous platform limestones and finally by the Early Miocene siliciclastic deposits of the Bifurto Formation. The uppermost tectonic units consist of chaotic clay of the Sicilide Units (Cretaceous-Oligocene) and the Eocene Albidona Formation of the Liguride Units. The Middle-Upper Miocene Gorgoglione Formation and Pliocene-Pleistocene shallow marine siciliclastic deposits cover unconformably this tectonic pile. In particular, the Contrada La Rossa area exposes an Eocene–Miocene turbidite succession deposited within thrust-sheet top basins, recording the progressive evolution of the southern Apennine chain (Fig 1C). This occurred during an early stage of oceanic subduction when the Liguride accretionary wedge formed on top of the W-dipping subduction of the Ligurian Tethys, and a second stage, related to the involvement of the Adriatic margin in the orogenic processes [24]. The turbidite sequences have been subdivided into the Albidona and the Gorgoglione Formations [25]. The Albidona Formation is bounded at the base by a low angle thrust contact, which is not exposed in the study area (Fig 3). Based on both outcrop and subsurface data, the age and stratigraphic organization of the Albidona Formation cropping out in the Contrada La Rossa area has been recently studied in detail [26]. Two main members have been recognized. The lower member, Ypresian/Lutetian in age, mainly consists of alternating lens-shaped sandstone beds and mudstone intervals. The occurrence of a pebbly mudstone, containing blocks of ophiolitic material, as well as igneous and metamorphic rocks, has been documented throughout the investigated area. Another associated lithology is represented by well-lithified, fractured marls varying in thickness from a few centimeters to tens of meters. The upper member, Priabonian/Barthonian in age, consists of alternating microconglomerates, sandstones, and clays, with intervals of marls and clayey marls forming meters-thick layers. The middle–upper Miocene Gorgoglione Formation mainly consists of meter-thick lens-shaped turbiditic sandstones, alternating with matrix-supported lens-shaped conglomerate/microconglomerate layers and clay-rich intervals. Sandstone and conglomerate layers are commonly organized in 10 to 50 m thick intervals, with minor centimeter- to decimeter-thick clayey intercalations. The marly intervals, commonly recognized in the Albidona Formation, are absent. The Gorgoglione Formation lies unconformably above the Albidona Formation (Fig 1C). According to [26], the Albidona Formation recorded multiple episodes of contractional deformation linked with its involvement first in the Ligurian accretionary wedge and later in the building of the southern Apennines chain. The Gorgoglione Formation shows lower deformation intensity related to the Apennine tectonic stages. Both successions are largely affected by Quaternary extensional and strike-slip faults connected to the formation of the high Agri Valley. The presence of marker beds in the studied successions, such as the pebbly mudstone interval and thick marly horizons, allow accurate mapping of the tectonic structures in the study area [26]. Three main fault sets, trending NW–SE, NE–SW, and nearly N–S, have been recognized (Fig 3). In particular, two roughly NE–SW-trending normal faults represent the most important tectonic structure in the study area and cause the exposure of progressively younger stratigraphic units towards the southeast (Fig 3). The western fault (the Figliarola Fault) separates the lower member of the Albidona Formation in the footwall from the upper member of the same formation in the hanging wall. The fault consists a NE-trending southern segment and a NNE-trending northern segment, separated by a NW-trending transfer fault. The electrical resistivity tomography (ERT) lines have been acquired along the southern segment of the Figliarola Fault and cross the transfer fault at its eastern termination. Along the Figliarola Fault, markedly different Albidona Formation lithologies can be observed. In particular, along the normal fault trace, vast outcrops of marls, with minor sandstone beds and clay, are present in the footwall, whereas the hanging wall is characterized by the occurrence of meter-thick lens-shaped bodies consisting of turbiditic sandstone and microconglomerate, intercalated within clay or marly clay. Most springs analyzed within this study are located along the northern segment of the Figliarola Fault, corresponding to the upper member of the Albidona Formation. The eastern fault (the Tempa del Vento Fault) separates the upper member of the Albidona Formation in the footwall from the Gorgoglione Formation in the hanging wall. The fault trace is frequently covered by Quaternary debris and landslide bodies, as observed in the Contrada la Rossa locality.

From the hydrogeological perspective, the study site belongs to the syn-orogenic turbidite series complexes, with an emphasis on the so-called arenaceous-conglomeratic complex, which is characterized by medium permeability when analyzed at a very small scale (1:250.000; [27]). Meteorological data from the closest meteorological station (N40.313888, E15.904444) that belongs to the regional agency for the environment protection (ARPAB, available at: http://www.arpab.it/opendata/q_aria_serie.asp), were downloaded to assess the climate of the area. The average temperature is 13.75°C and the cumulative annual precipitation is 701.46 mm, calculated for the period 2015–2019. According to the Köppen climate classification, the climate is Mediterranean (Csa), with dry and hot summers [28]. This result is consistent with the global classification reported in Beck et al. [29].

## Materials and methods

The multidisciplinary investigations were carried out in a two-step study. The first preliminary step was from May 2016 to April 2017 and the second step was from December 2017 to March 2019 and involved the same springs analyzed initially. The water sampling points were limited to those where isotope, chemical, and biomolecular analyses could be performed. The permits for sampling private wells were obtained by the wells’ owners, while for other sampling points (streams and public wells) no permission was required, since they were located over public areas and land.

### Geophysical investigations

Resistivity imaging was used to gain a better insight into the hydraulic framework of the system by comparing the results with those obtained by the geological survey (see Geological Setting). Geophysical data were collected using traditional and new generation instruments. The major target for investigation was the possible existence of widespread vertical heterogeneity, due to stress-release fracturing and/or rock alteration, which can enhance near-surface rock permeability and influence hydrogeological behavior.

Two different DC ERT systems were employed for collecting resistivity data (Table 1): a 48-electrode Syscal Pro System (SPS, IRIS Instruments, Orléans France) and an 8-unit MultiSource System (MSS, Multi-PhaseTechnologies, Sparks, Nevada, [30]). SPS data mostly targeted the near-surface geology while MSS data explored the subsurface at greater depths. The SPS is a geo-resistivity meter connected via multicore cable to 2 or more 24-electrode gathers. The console, through a series of internal switches, employs a dipole for injecting the current and it can read the potentials from up to 10 other dipoles simultaneously. The MSS is a new-concept geo-resistivity meter based on standalone independent units (transceivers), capable of both injecting current and measuring potentials. Each unit manages three electrodes, realizing all possible dipole combinations (1–2, 2–3, 1–3), and it communicates with the other units and with the command console via a 900 MHz radio signal. The MSS, besides its modularity and flexibility (e.g., ability to work around obstacles), can inject the current with multiple transmitting dipoles simultaneously [31]. Notably, with this design injection is limited to a single dipole per unit at a time and transmitting and receiving dipoles must belong to different units. There are no specific limitations on the number of simultaneous transmitters, but there is on the number of available units (currently up to 255). In this study case, the single- (1TX), double- (2TX), and quadruple- (4TX) transmitter modalities were designed and deployed in the field. Because the groundwater flows approximately from NW to SE, the resistivity profile was oriented almost perpendicular. The electrodes were laid along a gravel road approximately 1100–1120 m above sea level [m asl] (Fig 3). Spacing was set to 5.0 m and 25.0 m for the SPS and the MSS profile respectively. The SPS profile comprised a base sequence plus seven roll-along sequences with a 50% overlap while the MSS profile covered gathering the units in two blocks each comprising four units. The recording configuration in the SPS profile was Wenner-α and pole–dipole, resulting in a dataset of 8220 data points. MSS data were collected using the dipole–dipole configuration, and the dataset comprised 3540 measurements.

**Table 1:** Summary of SP and MS main acquisition parameters.

Feature	Array type	
	SPS	MSS
Array Type	Wenner α, Pole-dipole	Dipole-dipole
Total Length	1050 m	1175 m
Measurements	8220	3540
Electrode distance	5 m	25 m
Depth of investigation	∼60 m	∼285 m
Line Length	235 m	275m (pb)

Notes: pb = per block.

The two datasets were modelled and inverted in a 3D environment using the commercial software ERTLab Studio (Multi-PhaseTechnologies, Sparks, Nevada), based on a finite element modelling algorithm [32].

### Hydrogeological investigations

The discharge of three perennial and four seasonal springs (Fig 3) was measured monthly from December 2017 to March 2019, with an interruption between January and May 2018 due to the inability to access parts of the study area; although few springs were monitored from May 2016 to March 2019. The stream discharge was also measured in a couple of valleys to verify the presence of diffuse inflow from the surrounding groundwater (Fig 3).

### Water sampling

Stream and spring water sampling for stable isotope (oxygen and deuterium) analyses was conducted monthly from May 2016 to April 2017 and from late 2017 to March 2019. Sampling was performed once, in April 2016, for tritium analysis, to evaluate the mean residence time of spring waters during a recession period, with no influence related to possible rapid recharge. Rainwater samples for stable isotopes were collected monthly in two precipitation samplers (RWS1 at 1046 m. a.s.l. and RWS2 at 1290 m. a.s.l.; Fig 3) from May 2016 to April 2017. The rainfall was collected using 10 L polyethylene bottles containing approximately 300 mL of Vaseline oil to prevent evaporation. Oil contamination was carefully avoided by syringing the water samples out of the bottle.

Spring waters for chemical and biomolecular analyses were collected quarterly from June 2018 to March 2019 wherever possible.

Water samples for stable isotopes, tritium, chemical, and biomolecular analyses were stored in a refrigerated box and transported to the laboratory.

Electrical conductivity (EC) and temperature were measured *in situ* with portable equipment (multiparameter meter 9829, Hanna Instruments, Woonsocket, RI, USA).

### Chemical analysis

Water samples were filtered through 0.45 μm nylon filters (Whatman, Little Chalfont, UK). Samples devoted to metal analysis were acidified with ultrapure HNO_3_ to a final concentration of 0.6% (w/w).

Quantification of metal ions in groundwater samples was obtained by using an ICP-MS X Series II instrument (ThermoFisher Corporation, Waltham, MA, USA) equipped with an AS-500 autosampler (CETAC, Omaha, NE, USA). The operating parameters are described in the S1 Appendix. The absence of significant spectral interference was verified for all elements except for As and Fe for which an interference correction equation was applied.

The concentration of organic substances was determined using the permanganate oxidation method according to the UNI EN ISO 8467:1997.

Chlorine, fluorine, nitrate, and sulfate anions were determined according to standard methods 4500 Cl-F, 4500F-F, 4500 NO3-C, and 4500SO4-B. A brief description of the utilized equipment is reported in the S1 Appendix.

Validation was carried out according to the Eurachem guidelines by evaluating detection (LOD) and quantitation (LOQ) limits, linear range, precision, and trueness for each class of compounds.

Calibration curves were constructed for concentration levels in the ranges of 1–1000 μg L^-1^ for metal ions, 2–100 mg L^-1^ for chlorine, 0.5–3 mg L^-1^ for fluorine, 4–15 mg L^-1^ for nitrate, and 5–120 mg L^-1^ for sulfate. Lack-of-fit and Mandel’s fitting tests were performed to assess the goodness of fit and linearity, whereas the significance of the intercept (significance level 5%) was established by running a Student *t*-test.

### Statistical analysis of chemical data

Results of chemical analyses were investigated through a multivariate statistic approach, including only analytes above the LOD in at least two samples, to typify different patterns and groups among the chemical data. Prior to statistical analyses, the imputation of results under the LOD was performed using the zCompositions R package [33], adopting LOD from the above-mentioned methods. Successively, hydrochemical data were log-transformed due to differences in concentrations between major (e.g., SO_4_^2-^) and trace elements (e.g., Ni) [34]. Finally, principal component analysis (PCA) and permutational multivariate analysis of variance (PERMANOVA, Vegan package [35]; Euclidean distance, 999 iterations) were performed on the hydrochemical data using the R statistical software [36]. PERMANOVA was preferred to the multivariate analysis of variance (MANOVA) as the MANOVA assumptions were not met by the chemical data. Before performing PERMANOVA, the assumption of homogeneity of multivariate dispersions was verified using the betadisper function from the Vegan R package [35]. When PERMANOVA was significant and more than two groups were contrasted, a subsequent multilevel pairwise comparison was performed using the pairwise Adonis package [37] (Euclidean distance, 999 iterations, Hommel method for *p* values adjustment). For PCA, principal component (PC) selection was performed using the average eigenvalue approach [38]. The threshold for significance (alpha) was set to 0.05. Descriptive statistics of chemical data are reported using the form mean (standard deviation).

### Isotopic analyses

Stable isotope analyses (δ^18^O, δ^2^H) were performed at the Isotope Geochemistry Laboratory at the University of Parma (Italy), using a Delta Plus mass spectrometer (Thermo Fisher Scientific, Waltham, MA, USA) coupled to an automatic HDO device preparation system.

Analyses of ^3^H were performed according to the procedures provided by Water and Environment News No. 3 (1998) at the Isotope Geochemistry Laboratory at Trieste University, Italy.

### Next-Generation Sequencing (NGS) for bacterial community analyses

For bacterial community analyses, water samples (1 L) were filtered through sterile mixed esters of cellulose filters (S-Pak^TM^ Membrane Filters, 47 mm diameter, 0.22 μm pore size, Millipore Corporation, Billerica, MA, USA) within 24 h of collection. Bacterial DNA extraction from filters was performed using the commercial kit FastDNA SPIN Kit for soil (MP Biomedicals, LLC, Solon, OH, USA) and FastPrep^®^Instrument (MP Biomedicals, LLC, Solon, OH, USA). After extraction, DNA integrity and quantity were evaluated by electrophoresis in 0.8% agarose gel containing 1 μg/ml of Gel-Red^TM^ (Biotium, Inc., Fremont, CA, USA). The bacterial community profiles in the samples were generated by NGS (next generation sequencing) technologies at the Genprobio Srl Laboratory. Partial 16S rRNA gene sequences were obtained from the extracted DNA by polymerase chain reaction (PCR) using the primer pair Probio_Uni and Probio_Rev, targeting the V3 region of the bacterial 16S rRNA gene sequence [39]. Amplifications were carried out using a Veriti Thermal Cycler (Applied Biosystems, Foster City, CA, USA), and PCR products were purified by the magnetic purification step involving Agencourt AMPure XP DNA purification beads (Beckman Coulter Genomics GmbH, Bernried, Germany) to remove primer dimers. Amplicon checks were conducted as previously described [39]. Sequencing was performed using an Illumina MiSeq sequencer (Illumina, Hayward, CA, USA) with MiSeq Reagent Kit v3 chemicals. The fast files were processed using a custom script based on the QIIME software suite [40]. Paired-end read pairs were assembled to reconstruct the complete Probio_Uni/Probio_Rev amplicons. Quality control retained sequences with a length between 140 and 400 bp and mean sequence quality score > 20, while sequences with homopolymers > 7 bp and mismatched primers were omitted. To calculate downstream diversity measures, operational taxonomic units (OTUs) were defined at 100% sequence homology using DADA2 [41]; OTUs not encompassing at least two sequences of the same sample were removed. All reads were classified to the lowest possible taxonomic rank using QIIME2 [40, 42] and a reference dataset from the SILVA database v132 [43]. The sample biodiversity (alpha-diversity) was calculated with the Shannon index.

## Results

### Geophysical investigations

**Fig 4:**
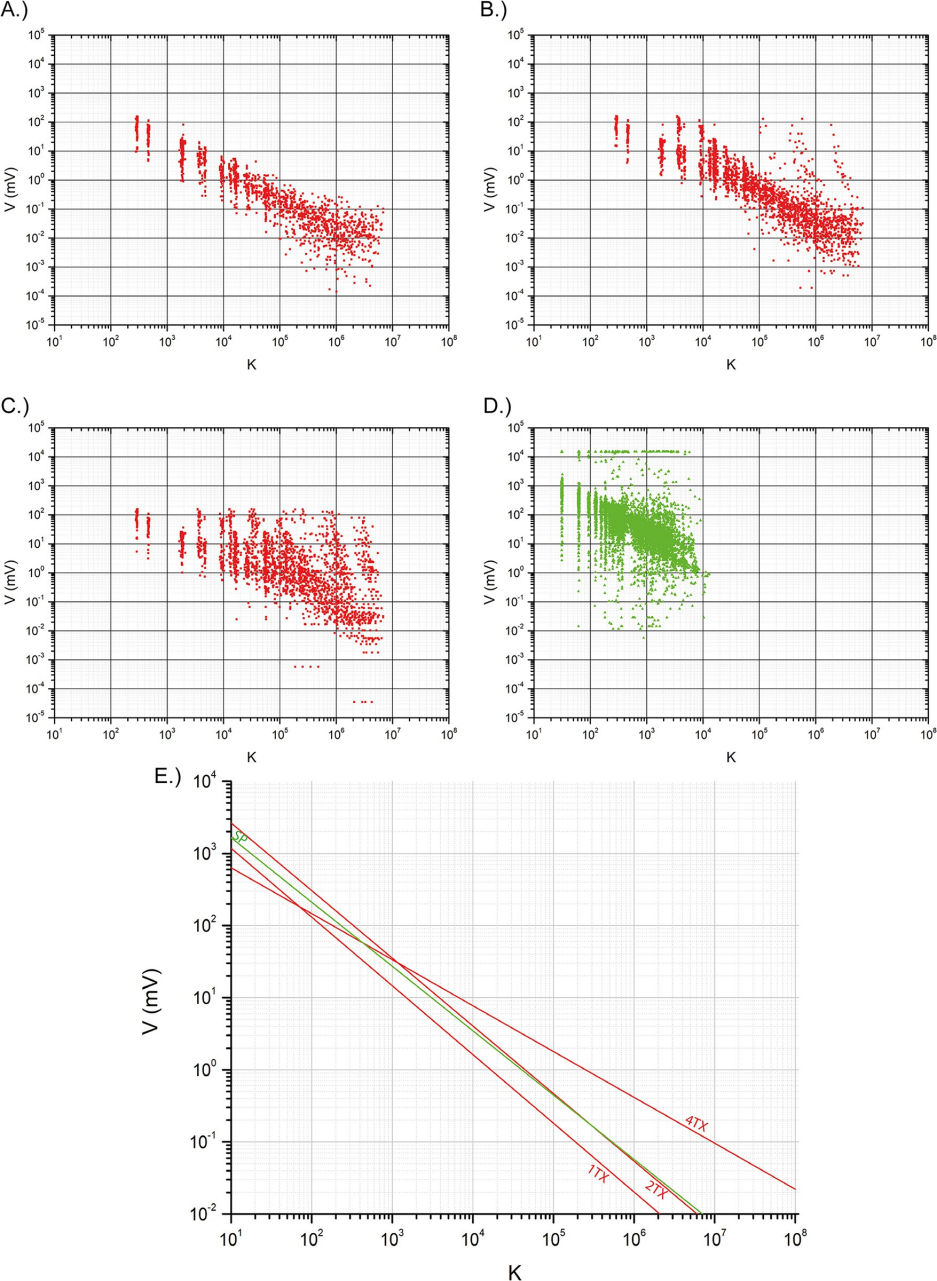
Comparison of recorded voltages (in mV) versus geometrical factor of the different datasets. (A) MSS-1TX; (B) MSS-2TX; (C) MSS-4TX; (D) SP; (E) regression functions for the different datasets.

A careful analysis of the measured potentials is crucial to estimate the uncertainty in assessing resistivity in the deeper geological layers. A rapid comparison between measured voltages and the geometric factor (K) (Fig 4) of each measurement showed that:

- data dispersion increased significantly switching from 1TX modality (R^2^ = 0.86) to 2TX (R^2^ = 0.74) and 4TX (R^2^ = 0.37). This effect was particularly evident for K > 10^4^–10^5^;
- the average voltage value of 6.95 mV in the1TX case reached 8.72 mV and 11.8 mV for the 2TX and 4TX cases, respectively. Even though this could be considered negligible, it assumed greater importance as the voltage increase for larger quadrupoles was up to two orders of magnitude (10^0^–10^2^ mV).

Direct comparison between MSS and SPS datasets is not straightforward because of the differences in the resistivity field geometry. Nevertheless, it should be noted that SPS data were very similar, in terms of dispersion, to the MSS-4TX dataset (R^2^ = 0.36); this similarity is associated with higher recorded voltage values (mean value of 588 mV). The main difference was in the fact that SPS data dispersion was quite large at K = 10^1^–10^3^ while MSS data, in the same interval, showed a well-defined trend. However, more data are required for a robust comparison between the two system performances.

As already mentioned, SPS and MSS measurements had different resolutions in the near-surface layers. SPS was preferable for exploring the uppermost 50–60 m of depth, while the MSS was crucial to map the depth continuity of the uppermost resistive bodies. The profiles were merged into a mosaic with the top and lower portions represented by the SPS and by the MMS profile, respectively.

**Fig 5:**
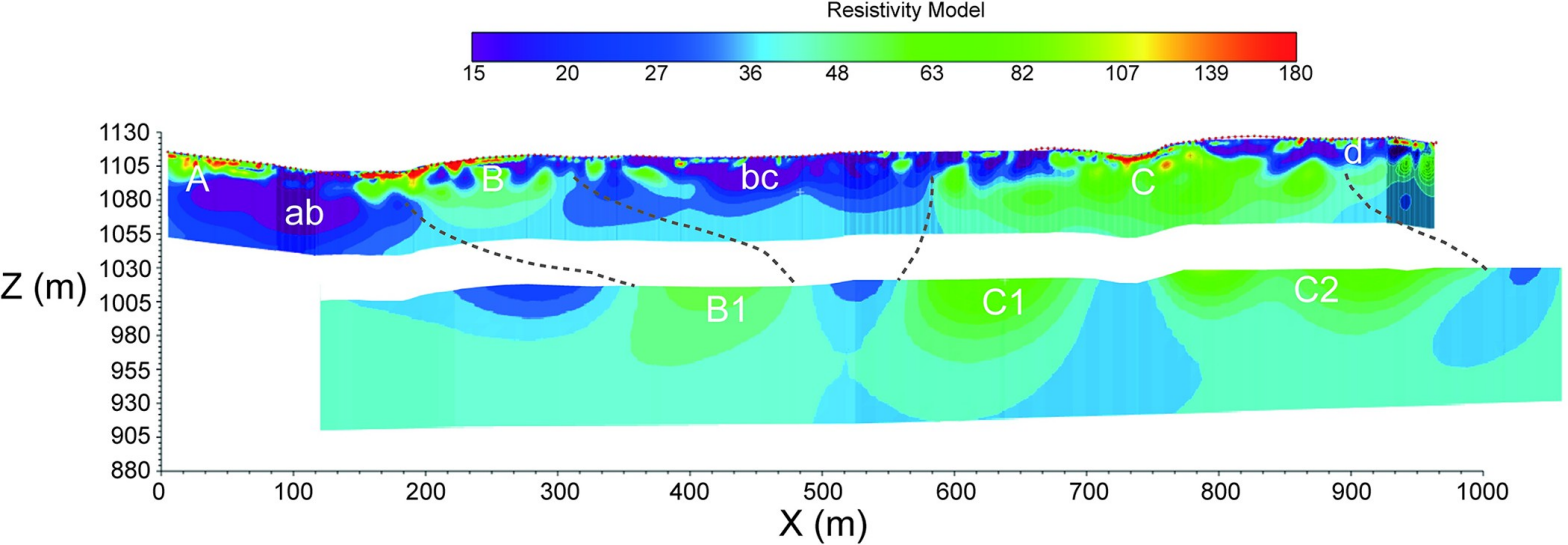
Mosaic of SPS (upper) and MSS (lower) ERT profiles.

For the SPS inverted profile, resistivity ranged from 15 to approximately 200 Ωm. Some highly resistive spots reached values slightly higher than 300 Ωm. Three different resistivity domains can be recognized as follows (Fig 5):

- Domain A, relatively resistive extending from the western edge of the section to approximately 80 m; a couple of lenticular structures (exhibiting resistivity of 250–300 Ωm) are visible within the uppermost 15–20 m;
- Domain B, which extends from 150 m to approximately 290 m. Three lenticular resistive bodies (250–300 Ωm) are visible; these bodies are thicker compared to domain A as they extend to 30–35 m depth;
- Domain C, which extends from 700 m to 800 m around “Fosso Lo Turno”. A resistive body (with values of approximately 300 Ωm) marks this section. The near-surface resistive bodies extend in depth, although resistivity appears to be slightly lower with values of 140–150 Ωm. The background resistivity in this domain is approximately 60–80 Ωm.

Subsurface resistivity in the other profile sections showed values more or less comparable with those of the background. Near-surface conductive domains ab, bc, and d are probably dominated by silty or clayey terrains (Fig 5).

The MSS profile (Fig 5) shows a homogenous resistivity background of approximately 35–40 Ωm interrupted by three large resistive bodies (B1, C1, and C2 in Fig 4). The base of these resistive bodies is located at approximately 150–180 m of depth. In the lower portion of the profile, the resistivity image appeared much smoother, and fell to background values.

Electrical signal penetration, as expected, was much larger compared to the SPS profile as it reached approximately 270–280 m below the surface.

The comparison of SPS and MSS data provided insight into data reliability. SPS and MMS correlated well, although with different degrees of resolution, in the eastern half of the profile. The near-surface resistivity field was complex in the western half of the profile and the correlation was poor, mostly due to the different electrode spacing.

The ERT line is oriented parallel to a major vertical fault (Fig 5), which makes geologic interpretation rather difficult. Unfortunately, the precise positioning of this structural element was not known at the time the survey was designed.

The geology consists of folded alternating of soft pelite and moderately hard sandstone layers. These layers are also differentiated by resistivity (Ω) as the pelite is quite conductive while the sandstone is more resistive. The sandstone layers are thinner, as they are often embedded in a pelite matrix, thus affecting the resistivity field that appears smoothed at large depths. Furthermore, sandstones are organized in lenticular bodies. These bodies exhibited poor lateral continuity resulting in marginal overall subsurface resistivity values.

Resistive anomaly A is probably related to a conglomerate body outcropping just south of the survey line, while anomaly B, beginning on the eastern side of “Fosso della Rossa” and extending eastwards, could be the signature of marly layers and sandstone units of the Albidona Formation. The marly layers dip NE and are located north of the survey line, while the sandstone units are almost vertical and oriented perpendicular to the survey line. The near-surface anomaly B could be correlated to the deeper resistive (> 60 Ωm) body B1. Outcrop bedding geometry is not consistent with this hypothesis, but some changes in bedding geometry could be expected at greater depths. Strata are probably heavily folded and overturned near the fault plane.

Conductive units AB, BC, and D are likely comprised of the Albidona Formation pelite layers.

Resistive anomaly C with its associated lobes C1 and C2 is the most prominent electrical feature along the survey line. It may be related to the presence of lenticular sandstone bodies embedded in the pelitic matrix of the Albidona Formation. Strata bedding is almost vertical and perpendicular to the survey line, which may explain the persistence of relatively high resistivity at depth.

An overall analysis of the resistivity image suggests that the deeper subsurface section is mostly comprised of pelites with a high fraction of clay minerals for the first half of the survey line. These deposits, based on the resistivity field (15–30 Ωm), are likely characterized by very low to low permeability. In the second half of the survey line, the scenario is slightly different; in-depth resistivity stays at relatively high values of 70–80 Ωm for much of the subsurface. The value itself is not as high, but it is a clear indication that there are several relatively resistive units in a pelitic background. Several lenticular bodies are mostly comprised of arenites. are visible in the geological map (Fig 3). Shallow resistive nuclei are also visible in the shallow layers of the resistivity profile (Fig 5). These resistive nuclei are probably to be correlated with the lenticular bodies. The permeability of these deposits, based on the sole resistivity field, is expected to be notably higher than the average permeability of the western half of the survey line, thus emphasizing the heterogeneity of the investigated system.

### Hydrogeological investigations

**Fig 6:**
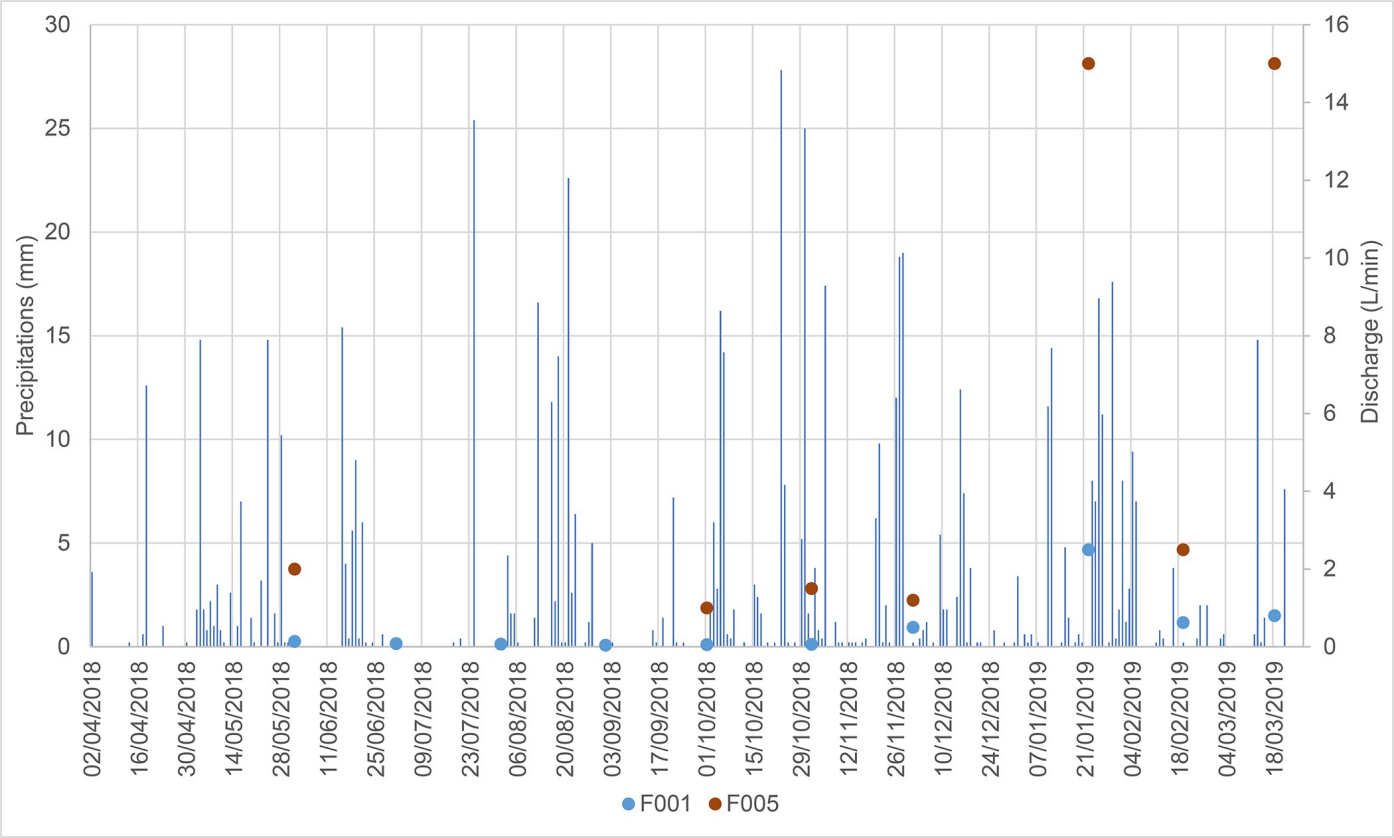
Discharge measured at F001 (upstream) and F005 (downstream) along a single stream.

The spatial distribution of springs was clearly affected by both geological and morphological features, suggesting that lithological heterogeneity is only one of several factors influencing groundwater outflow at the study site (Fig 3). Diffuse springs were also detected along the studied streams, and a significant increase in discharge was measured throughout the observation period (up to several L/min; see example in Fig 6).

**Fig 7:**
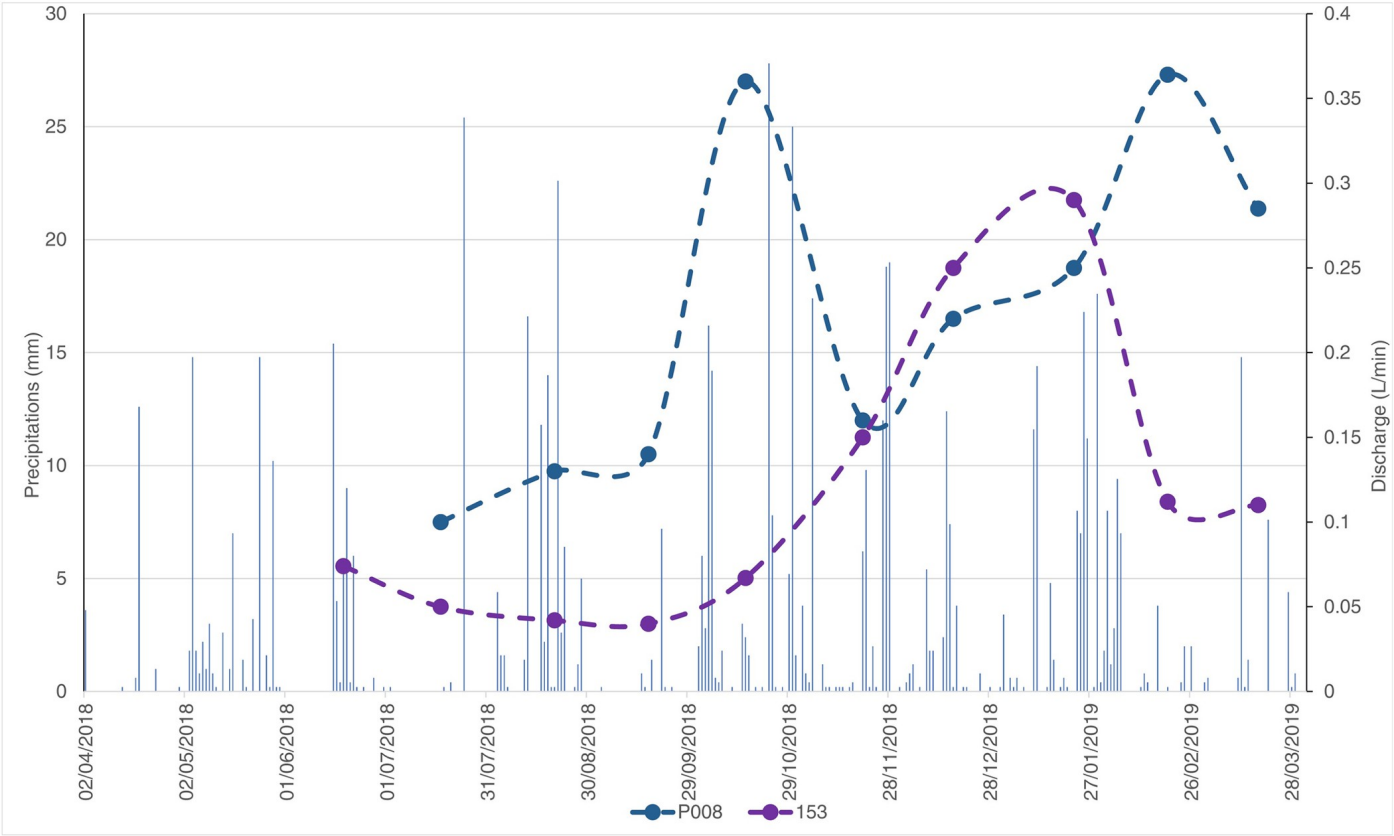
Examples of spring hydrographs (dates are given in month/day/year).

The spring discharge varied throughout the year from 0 (seasonal springs) to 0.5 L/min. Overall, two main regimes were observed: (i) a smoothed one, characterized by a prolonged recession in summer and early autumn, and a progressive increase in discharge during late autumn and winter (see spring 153 in Fig 7); and (ii) an irregular one, characterized by faster response to precipitation events, with a sequence of different increasing and decreasing phases over the hydrologic year (see spring P008 in Fig 7). In both cases, a recession occurred during two rainfall periods, probably due to a negligible effective infiltration (and groundwater recharge) caused by the aforementioned precipitations. However, a more reliable interpretation of this phenomenon could be obtained through a continuous discharge monitoring of such irregular springs.

### Physicochemical features

**Fig 8:**
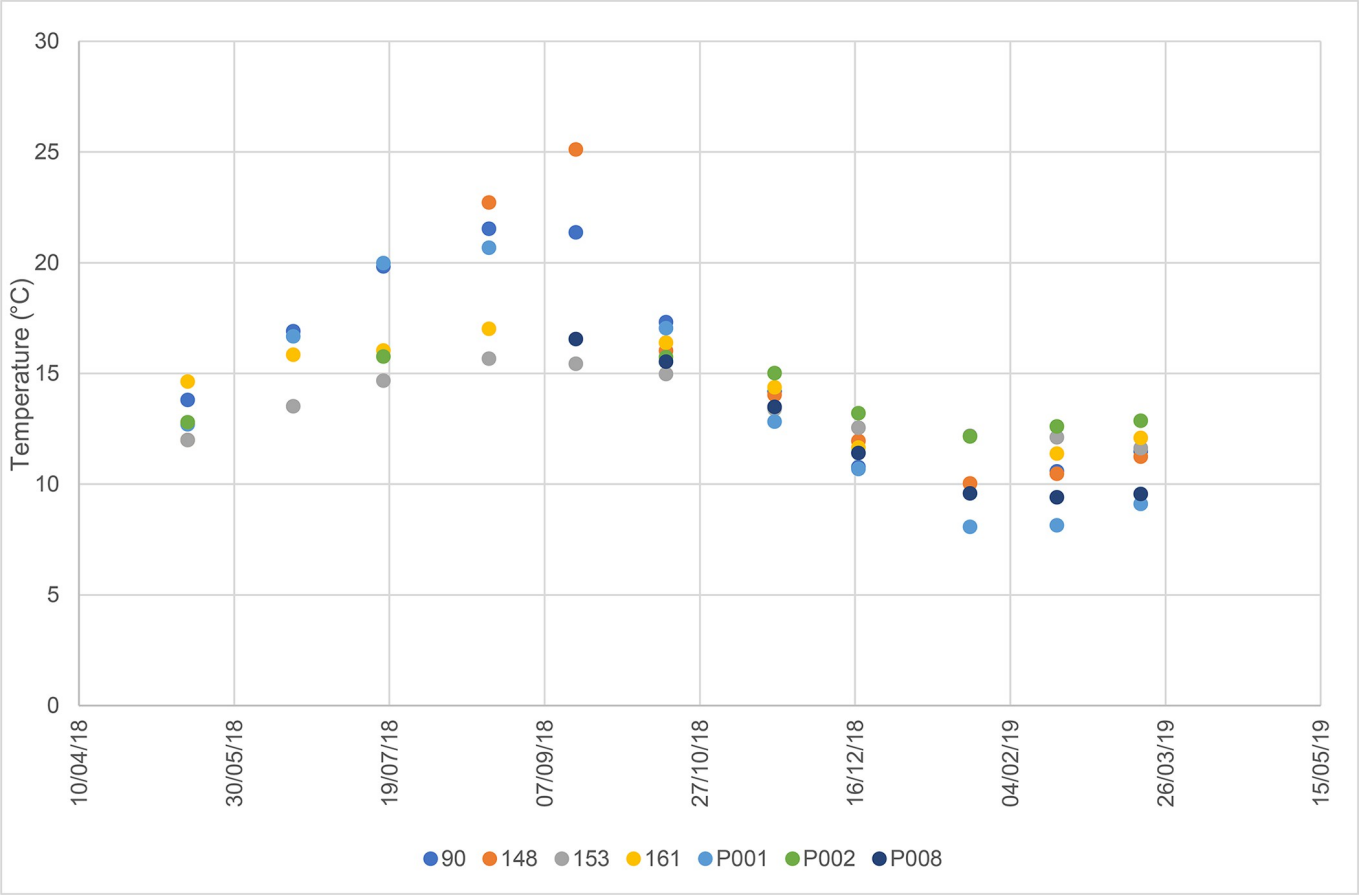
Comparison between thermal fluctuations in spring waters. Dates are given in day/month/year.

Among the physicochemical parameters, water temperature provided interesting information. All springs (both perennial and seasonal) were characterized by the same temporal variation in water temperature (Fig 8), apart from the type of hydraulic regime (smoothed or irregular). They showed significant temperature fluctuations over time (up to 12° C), with maximum values in summer and minimum ones in winter, suggesting that the observed springs are fed by shallow groundwater flowing within the heterothermic zone.

**Fig 9:**
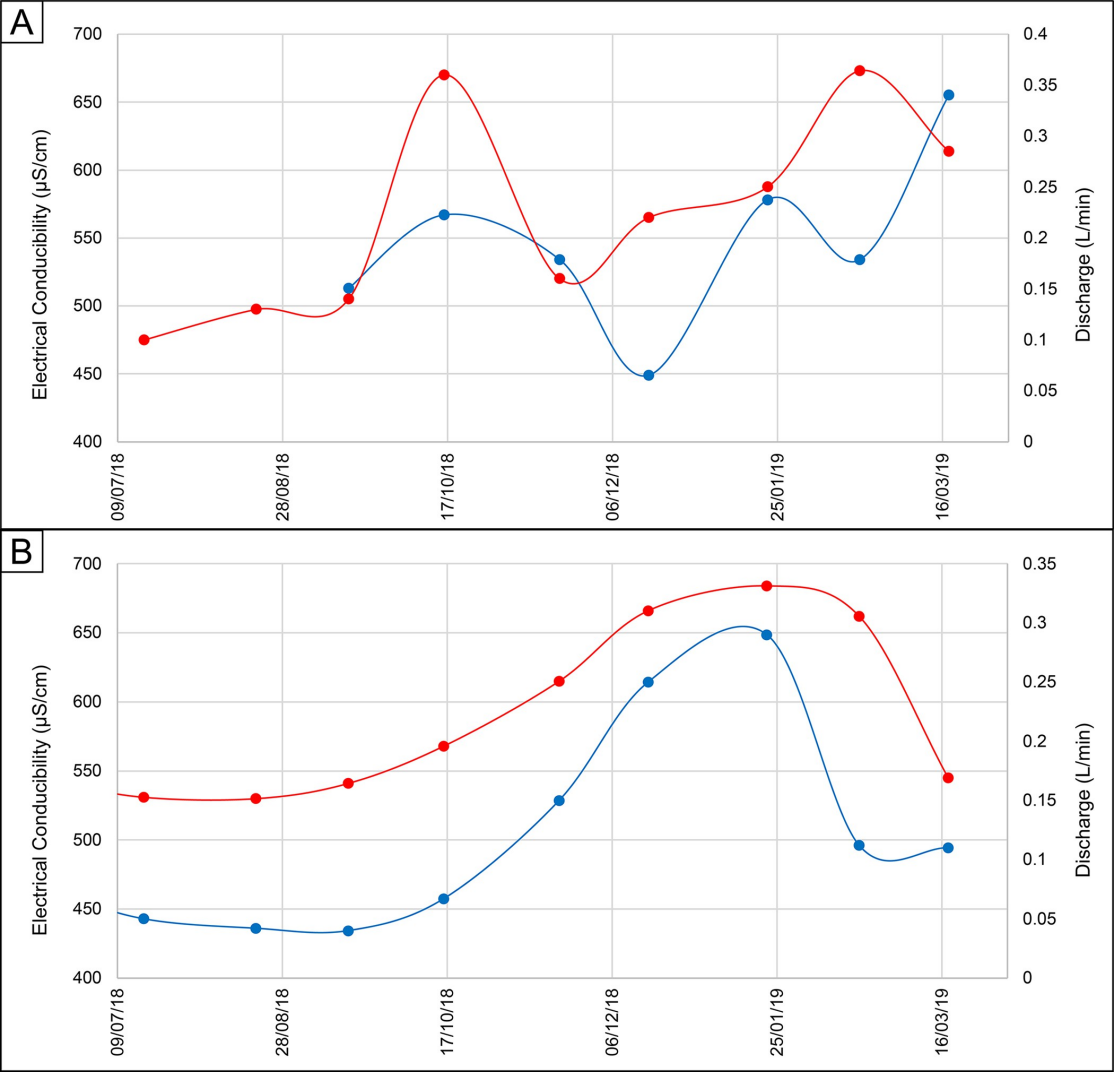
Examples of EC fluctuations (red line) vs. discharge (blue line). Graph A) represents spring P008 and graph B) in spring 153. Dates are given in month/year.

Unlike water temperature observations, the water EC varied over time in a different manner, depending on the type of hydraulic regime. In detail, the springs characterized by the smoothed-type regime showed a direct relationship between discharge and EC (Fig 9B). This observation suggests that the increase in salinity is influenced by the hydraulic head rise within the aquifer system due to the arrival of fresh infiltration water on the groundwater surface, and the consequent mobilization and outflow of groundwater characterized by higher mean residence time. Nevertheless, springs characterized by the irregular hydraulic regime, such as the P008, showed a more articulated variation in EC over time, and often an inverse relationship between EC and discharge (e.g., December 2018 and February 2019 in Fig 9A).

**Fig 10:**
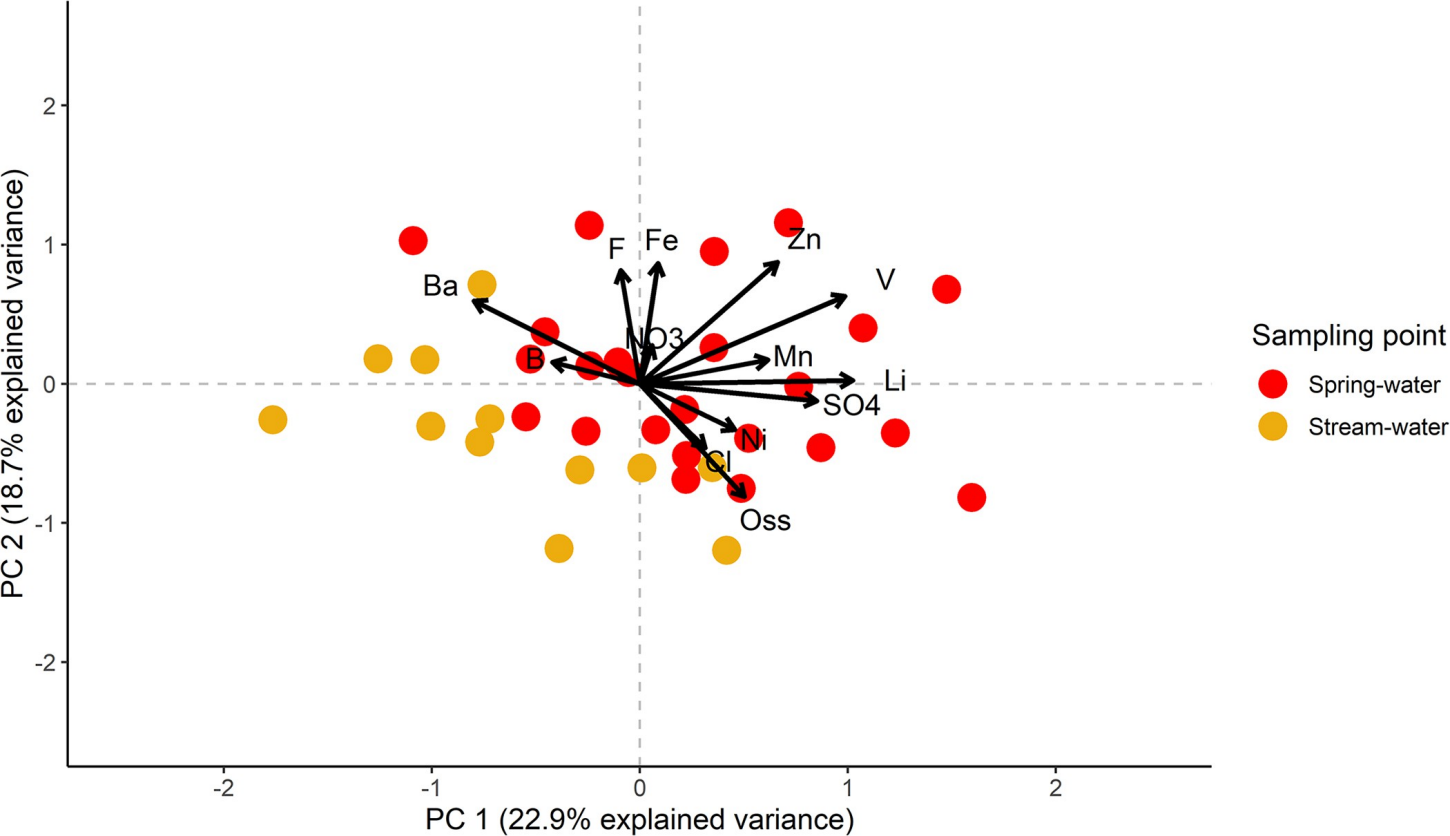
Plot of PCA1. Acronyms are used for permanganate oxidation (Oss), NO_3_^-^ (NO3), Cl^-^(Cl), F^-^ (F), and SO_4_^2-^ (SO4).

For the statistical analyses of the hydrochemical data, the first PCA (PCA1) was applied to the whole dataset (Fig 10) of chemical results. PCA1 eigenvalues, total variance, and factor loadings are reported in Table 2.

**Table 2:** PCA eigenvalues, total variance, and factor loadings of PCA1 and PCA2.

	PCA1					PCA2			
	PC1	PC2	PC3	PC4	PC5	PC1	PC2	PC3	PC4
**Eigenvalue**	2.978	2.430	1.627	1.464	1.279	3.112	2.558	2.034	1.440
**Total variance**	22.91%	41.59%	54.11%	65.37%	75.21%	23.94%	43.62%	59.26%	70.34%
**Ba**	-0.797	0.594	-0.062	0.316	-0.172	-0.836	-0.062	-0.115	0.404
**B**	-0.419	0.155	-0.768	0.587	0.499	-0.577	-0.593	-0.111	0.457
**Fe**	0.088	0.865	0.097	-0.498	0.474	-0.490	0.539	0.527	0.089
**Li**	1.024	0.025	-0.646	0.200	-0.105	0.834	0.102	-0.208	0.574
**Mn**	0.616	0.171	0.497	0.578	-0.147	0.198	0.633	-0.599	-0.152
**Ni**	0.457	-0.330	-0.436	-0.572	-0.114	0.657	-0.064	0.388	0.322
**V**	0.986	0.628	0.272	0.124	0.005	0.021	1.085	-0.136	0.209
**Zn**	0.662	0.873	0.412	0.106	-0.134	-0.138	1.066	-0.042	0.132
**Oss**	0.503	-0.810	0.277	-0.064	0.396	0.699	-0.016	-0.247	-0.478
**Cl** ^**-**^	0.314	-0.461	0.388	-0.364	0.712	0.704	0.109	0.645	0.286
**F** ^**-**^	-0.091	0.813	-0.332	-0.331	0.682	-0.561	0.197	0.604	0.332
**NO**_**3**_^**-**^	0.059	0.275	-0.325	-0.839	-0.655	0.369	0.002	0.979	-0.303
**SO**_**4**_^**2-**^	0.851	-0.123	-0.749	0.254	0.049	0.580	-0.223	-0.296	0.739

**Fig 11:**
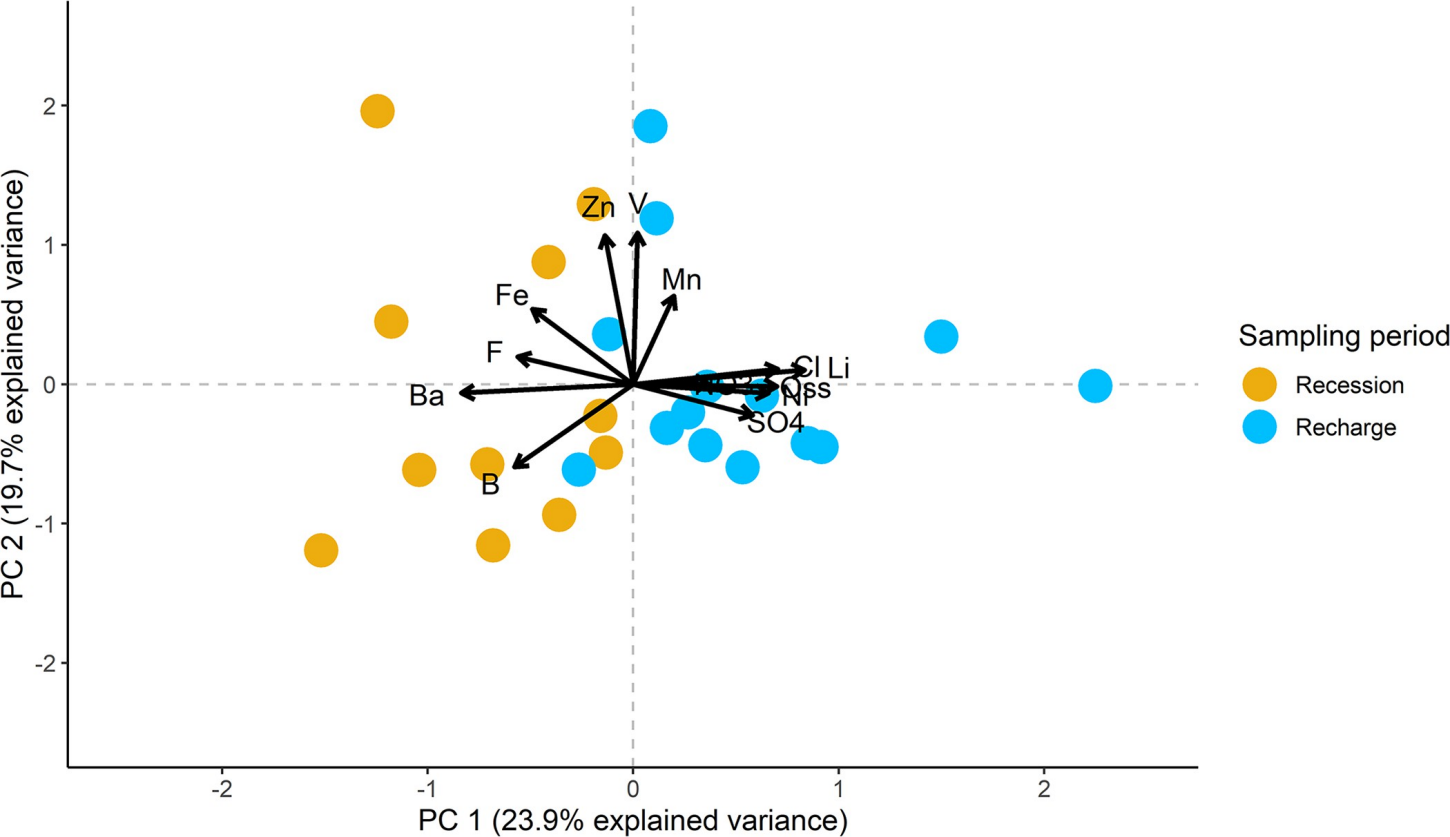
Plot of PCA2 (only spring water samples) according to sampling period. Acronyms were used for permanganate oxidation (Oss), NO_3_^-^ (NO3), Cl^-^(Cl), F^-^ (F), and SO_4_^2-^ (SO4).

Looking at the PCA1 plot, the first PC has a stronger relationship with Li, V, and SO_4_^2-^ (positively related), and Ba and B (negatively related). The second PC has a stronger relationship with F, Fe, and Zn (positively related) and permanganate oxidation (negatively related). Based on Fig 10, a good separation exists between surface and spring water samples. Dissimilarities among these groups appear to be driven primarily by PC1 and only partially by PC2. These differences were tested using PERMANOVA, which showed that the differences were significant, with F(1, 35) = 4.9995, *p* = 0.002, confirming the interpretation of PCA1 plot. To better investigate the pattern in groundwater samples, a new PCA (PCA2) was performed on a subset of the original data, including only spring water samples. The PCA2 results are reported in Table 2 and Fig 11.

**Fig 12:**
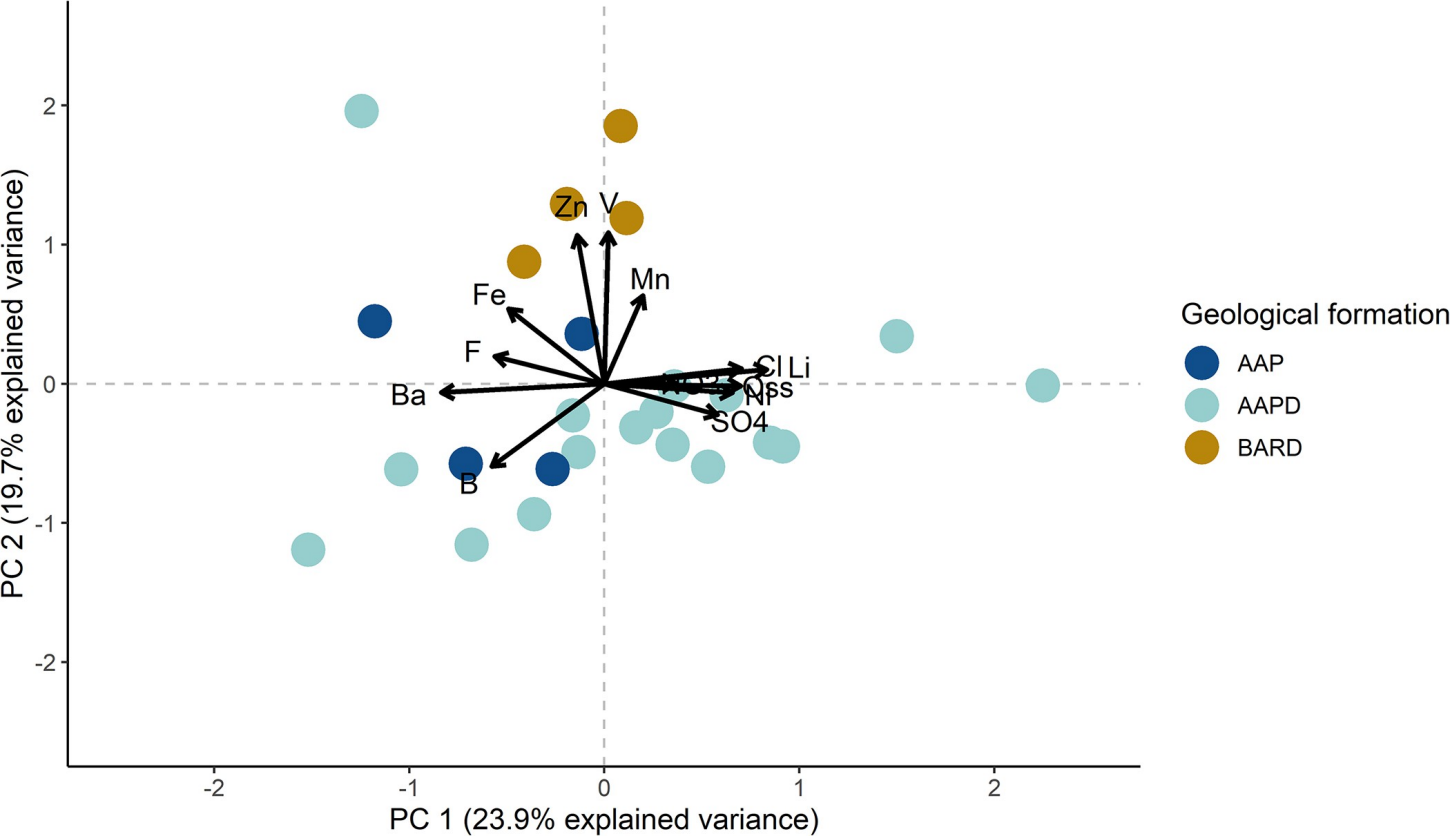
Plot of PCA2 (only spring water samples) according to geological formation. Acronyms are used for permanganate oxidation (Oss), NO_3_^-^ (NO3), Cl^-^(Cl), F^-^ (F), and SO_4_^2-^ (SO4).

In Fig 11, PC1 has a stronger relationship with Li, permanganate oxidation, Cl^-^, SO_4_^2-^, and Ni (positively related) and Ba, F^-^, Fe, and B (negatively related). PC2 is strongly related to V, Zn, and Mn (positively related) and B (negatively related). Samples from recession and recharge periods are generally plotted on different sides of the graph, showing differences better explained by PC1. According to PERMANOVA, the difference between the recession and recharge periods was significant, with F(1, 23) = 3.6427, *p* = 0.007. In PCA2, PC2 explained much of the total variance (19.7%) and factor loadings oriented along this component were perpendicular to those along PC1, underlying the low correlation between them. Additionally, other possible differences in the PCA2 plot were explored using the geological features of the study area (Fig 12).

As reported in Fig 12, the hydrochemical differences among samples can vary according to the Albidona Formation member, as highlighted also by PERMANOVA, with F (2, 22) = 4.417, *p* = 0.003. In particular, samples from spring S148 located in the Albidona *C* (BARD) were different from those located in Albidona *D* (AAPD) with F(1) = 7.554, adjusted *p* = 0.009 and Albidona B/C (AAP) with F(1) = 4.936, adjusted *p* = 0.040. On the contrary, hydrochemical differences between AAPD and AAP were not significant, with F(1) = 0.739, adjusted *p* = 0.576. Comparing the few mineralogical aspects of the geological formations available for the area (unpublished data) with the hydrochemical characteristics of springs water, a good accordance is found.

Although NO_3_^-^ does not appear to be pivotal in the PCA outputs (Table 2), its concentrations are better explored spatially (Table 3). Moreover, the presence of farms and related agricultural practices could represent a source of contamination and lead to higher NO_3_^-^ values locally compared to the environs, where more natural areas were observed (e.g., meadows and woods).

**Table 3:** Nitrate concentrations showing mean and standard deviations (SD) in the investigated springs (mg/L NO_3_^-^).

	Sep-18	Dec-18	Mar-19	Jun-19		
**P001**	< LOD	7.2	2.60	0.28	3.36	3.52
**P002**	< LOD	30	26.00	< LOD	28.00	2.83
**P008**	6.90	6	8.00	5.00	6.48	1.28
**S148**	7.00	< LOD	1.20	6.70	4.97	3.27
**S153**	8.50	< LOD	12	9.00	9.63	1.60
**S161**	< LOD	< LOD	1.4	0.39	0.90	0.71
**S90**	1.60	3.9	3.49	2.20	2.80	1.08
	6.00	11.22	7.81	3.93	**Mean**	
	3.02	10.66	8.94	3.56		**SD**

In groundwater, during the investigated period all the springs were characterized by a relative low concentration of NO_3_^-^, corresponding to 7.20(7.49) NO_3_^-^ mg/L. Spring P002 was characterized by the highest value, namely 28.00(2.83) NO_3_^-^ mg/L, while the lowest was observed in spring S161, with a concentration of 0.90(0.71) NO_3_^-^ mg/L. Within surface waters, a significant difference between F001 and F002 was observed, 1.00(0.28) NO_3_^-^ mg/L and 8.30(2.37) NO_3_^-^ mg/L, respectively.

### Isotope geochemistry

The following local precipitation regression line was calculated:

$$\delta^{2}H=[6.48\;(\pm 0.66)\;\delta^{18}O+4.58\;(\pm 5.19)]‰$$ (1)

s(yx)=4.56‰,n=21p(A=0)=0.39

where *s(yx)* is the standard error of regression, *n* is the number of data pairs, and *A* is the intercept of the regression line. This meteoric water line is in agreement with those calculated by several authors in southern Italy, e.g. [8, 44–46].

**Fig 13:**
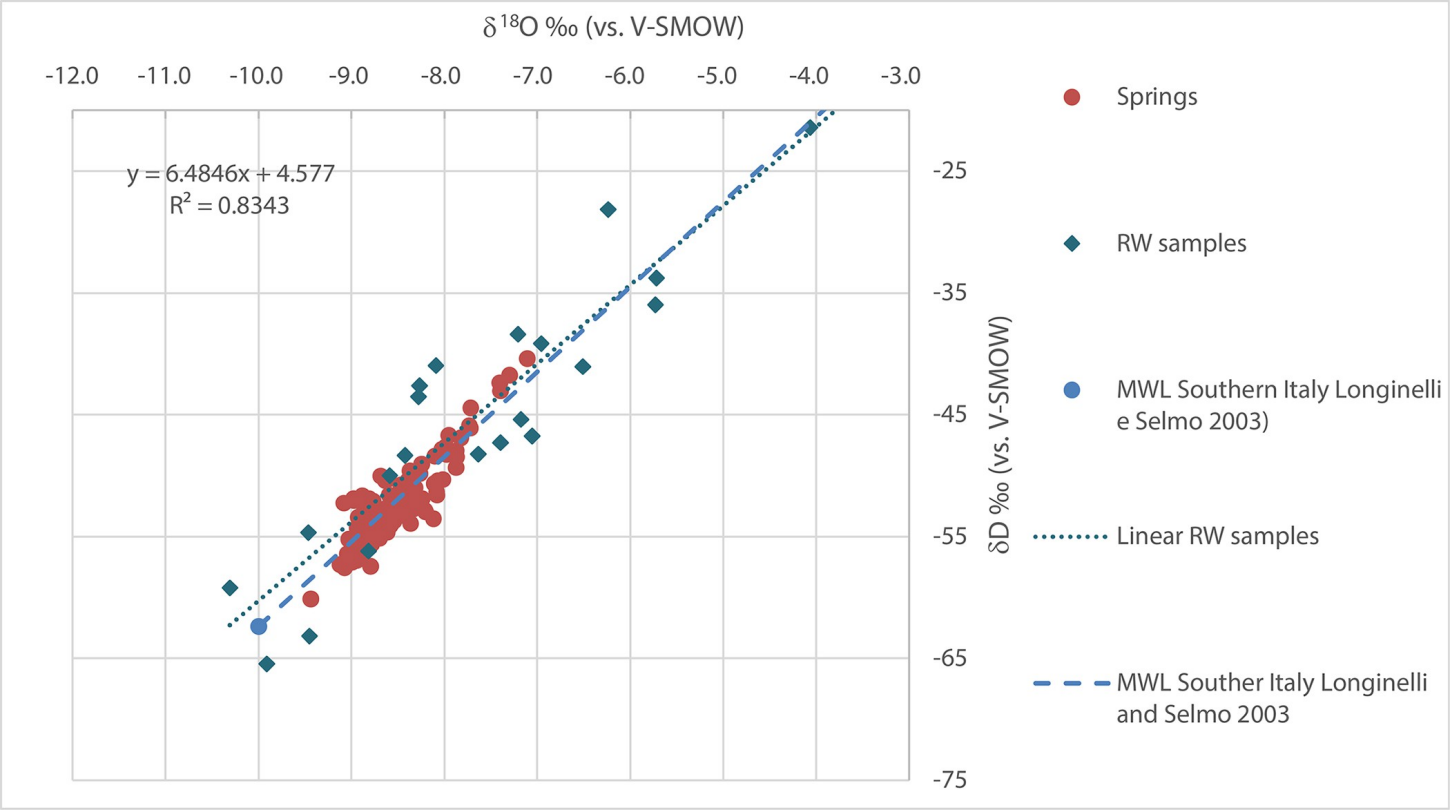
Relationship between δ^2^H and δ^18^O. The red dots represent the investigated waters and the black dots symbolize the isotopic signature of local rainwater. The dashed black line shows the local meteoric water line.

The samples of all spring and stream waters fall close to line (1), indicating a prevalent meteoric origin (Fig 13).

The isotopic signature of stream water samples varied significantly over time (e.g., standard deviation = 0.44‰ for δ^18^O and 3.88‰ for δ^2^H in F001), according to the hypothesized mixing ratio between ground and runoff waters.

**Fig 14:**
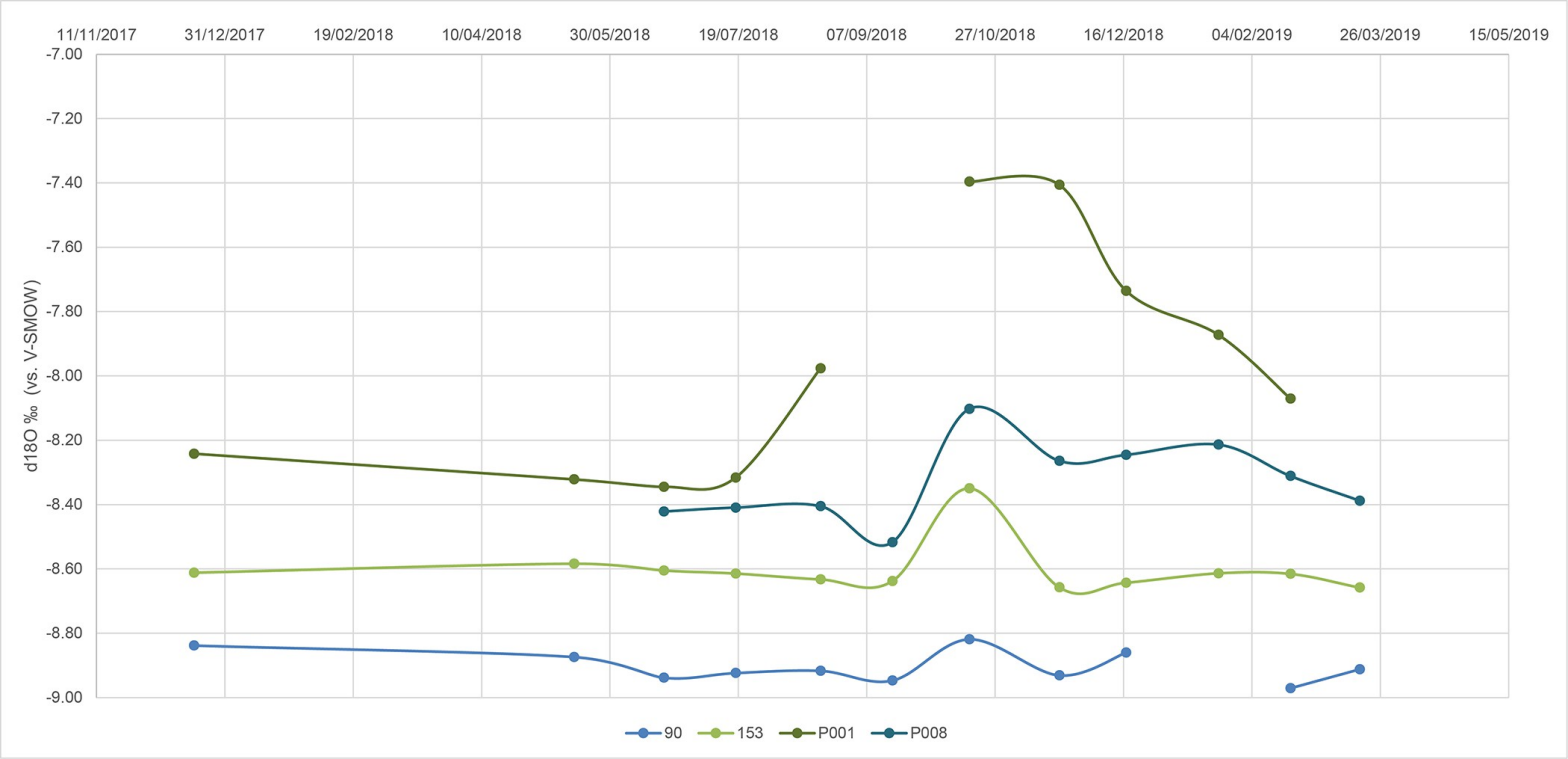
Variation in the isotopic signature of spring waters over time.

Differently, the isotopic content of spring water samples varied little over time in most of the analyzed springs (see, for example, spring 90 in Fig 14). Actually, their isotopic variations were lower than 2*u*, where *u* is prediction uncertainty for δ^18^O and δ^2^H data. In some cases, such as spring P001, a significant increase in δ values were observed, especially in autumn, due to a water influx characterized by higher δ values (summer rain). The contribution to the springs by the summer rains was higher and earlier at P001 than at the other springs depending, probably, on the higher permeability of the landslide body within which it is located (Fig 3).

Similar behavior was observed when analyzing other springs, such as 153 and P008. The pre-autumn isotopic spring values were due to different elevations of the rain recharge areas.

Tritium content detected during the recession (with no influence related to possible rapid recharge) ranged between 2.5 ± 0.6 and 3.9 ± 0.7 TU (Table 4), suggesting the coexistence of both more (e.g., 2.5 ± 0.6 TU in P002-waters) and less prolonged groundwater pathways (e.g., 3.9 ± 0.7 TU in S90-waters) within the studied system. When compared with the tritium content recently detected in rainwater collected in southern Italy (4.5 TU in [8]; 5.0 TU in [20]; 6.2 to 10.8 TU in [47]), they indicate approximately 2.5–24.5 years of ground residence. In detail, no significant differences were observed among the whole range of spring (2.5 ± 0.6 to 3.9 ± 0.7 TU) and stream waters (2.6 ± 0.6 to 3.8 ± 0.7 TU), indicating that the streams were mostly recharged by the springs, at least in the sampling period.

**Table 4:** Tritium content in spring and stream water samples.

Spring or stream water sample	Tritium content (TU)
F001	3.8 ± 0.7
F002	3.4 ± 0.7
F003	3.3 ± 0.6
F005	2.6 ± 0.6
S90	3.9 ± 0.7
S148	2.9 ± 0.6
S153	3.8 ± 0.7
S161	2.7 ± 0.6
P001	2.7 ± 0.6
P002	2.5 ± 0.6

Spatially, the water samples collected within the downgradient portion of the study area were mainly characterized by the lowest tritium content (2.5 ± 0.6 to 2.9 ± 0.6 TU in S148, S161, P001, P002; Table 4), according to an overall convergence between the groundwater pathway and the local morphology.

### 16S ribosomal RNA gene Next-Generation Sequencing (NGS)

Water samples for microbiological investigations were collected in June 2018, September 2018, January 2019, and March 2019. Data from some seasonal springs are missing for certain sampling campaigns. Table 5 reports the number of sequences produced by MiSeq runs for the examined springs. The 16S rRNA gene sequences generated in this study have been deposited in the NCBI sequence read archive (SRA) under the accession number PRJNA722990 in the BioProject called “Contrada la Rossa”.

**Table 5:** Number of 16S rDNA sequences obtained after NGS analysis for the four sampling campaigns.

Sample	Final Read Number			
	Sampling Campaigns			
	June 2018	September 2018	January 2019	March 2019
90	n.a	n.a	n.a	62209
148	n.a	58457	62154	60968
153	82371	66310	40723	45281
161	73251	n.a	n.a	62772
F001	56151	63434	81346	62606
F002	70491	n.a	74705	69228
F003	n.a	n.a	n.a	56124
F005	80397	n.a	116935	60630
P001	n.a	n.a	69912	71780
P002	n.a	n.a	77086	45717
P008	n.a	n.a	63778	n.a

n.a. represents not available samples.

The rarefaction analysis highlighted a greater microbial diversity in both spring and stream waters and the Shannon index was determined for all samples and ranged from 2.58 to 9.98 (Table 6; S2 Fig).

**Table 6:** Microbial diversity estimates obtained from the Shannon index.

Sample	Shannon Index			
	Sampling Campaigns			
	June 2018	September 2018	January 2019	March 2019
90	n.a	n.a	n.a	6.13
148	n.a	3.86	2.58	2.79
153	8.37	6.07	7.85	6.15
161	6.91	n.a	n.a	7.01
F001	5.64	5.38	9.44	8.08
F002	8.33	n.a	9.66	7.90
F003	n.a	n.a	n.a	7.08
F005	8.76	n.a	9.30	6.71
P001	n.a	n.a	9.05	7.61
P002	n.a	n.a	9.98	3.27
P008	n.a	n.a	7.31	n.a

n.a. represents not available samples.

NGS results revealed the presence of twenty different phyla: the most abundant were *Proteobacteria*, *Bacteroidetes*, *Firmicutes*, *Cyanobacteria*, *Actinobacteria*, and *Patescibacteria*. In particular, *Proteobacteria* was predominant in most samples, with relative abundance values ranging from 33.52% to 96.41%. When analyzing microbial community composition over time at this level (for stream water examples F001 and F005, and spring water sample 153), this phylum was always the most represented, except for in June 2018 in spring 153 (where *Proteobacteria* and *Firmicutes* were found at similar levels) and for the September 2018 in F001 (where the most abundant phylum was *Bacteroidetes*) (S2 Fig).

At the genus taxonomic level, greater detail is given for the microbial community composition of the springs and stream. The waters were characterized by partially different microbial communities that varied over time, in part due to seasonal climate changes. *Rhodoferax*, *Acinetobacter*, *Pseudomonas*, *Flavobacterium*, *Sideroxydans*, and *Hydrogenophaga* were the most common genera found (S3 Fig).

The presence of some genera like *Lactobacillus*, *Streptococcus*, *Bifidobacterium*, *and Bacteroides* in springs 148 and 153 supports the hypothesis of possible microbial pollution of fecal origin, in agreement with the presence of pasture and utilization of manure in local agricultural practices (e.g., [48]). The greatest abundance of these genera was recorded in June and September 2018 whereas in January and March 2019 these taxa were found at lower percentages. However, denitrifying bacterial genera, such as *Rhodoferax* and *Pseudomonas*, were detected in all water samples, therefore suggesting that the variation in nitrate concentrations within the study site could also be influenced by biological factors.

### Hydrogeological conceptual model

Geological and geophysical surveys confirmed the significant permeability distribution heterogeneity of the studied medium according to the geological features of other turbidite sequences investigated from the hydrogeological point of view. Furthermore, both surveys showed no vertical heterogeneity related to stress-release fracturing and/or rock alteration, thereby excluding the presence of a shallower and widespread horizon characterized by higher rock permeability in the near-surface medium. Based on these findings, we can exclude the presence of a perched groundwater (ascribable to the so-called “shallow system” found by [4] (within the Northern Italian Apennines)) due to the permeability contrast with depth, and all the analyzed springs are fed by a unique groundwater system flowing in a low-permeability continuum (according to the tritium content in spring waters), at the basin scale. The basin scale hydraulic continuity is probably enhanced by the fracture network associated with faults and folds, which interrupt the continuity of the low-permeability layers (low aquitard integrity, *sensu* [49]), thereby excluding an aquiclude role played by each of these layers. This hypothesis agrees with the connection between fluid flow and fault/fracture zones associated with thrust folds observed by other authors (e.g., [50]).

**Fig 15:**
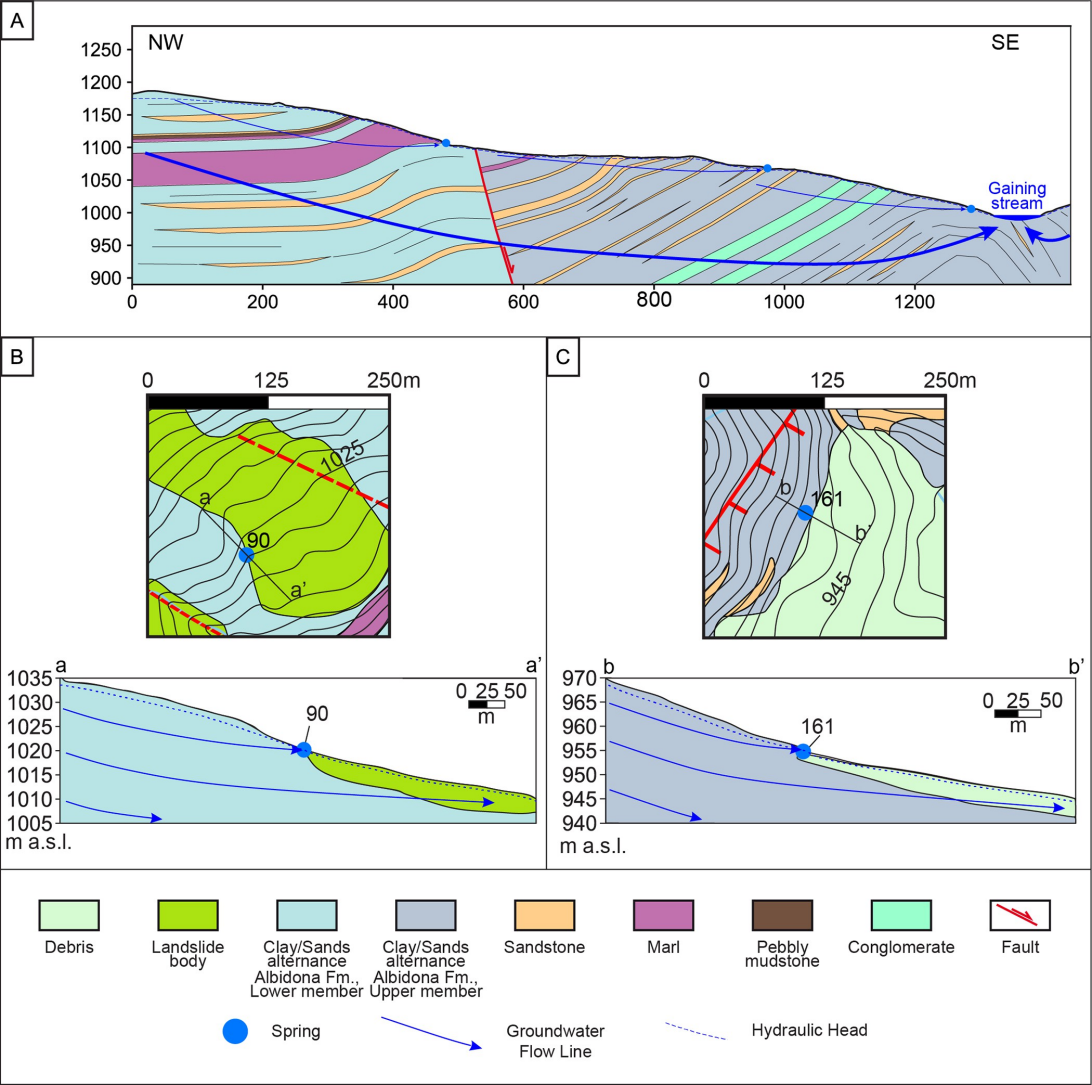
Hydrogeological conceptual model.

The spring’s location is probably influenced by the relationship between the dip and slope angles, according to [10]. Overall, the spatial distribution of springs and the diffuse groundwater outflow along them suggest an overall adaptation of the potentiometric surface to the aquifer morphology. In this scenario, the thin unsaturated zone and the spatial variations in the topographic gradient cause the shallower groundwater to flow out at the multiple intersections between the phreatic surface and the topographic one (Fig 15). In greater detail, we hypothesize that the relatively high hydraulic gradient, comparable to the topographic one, is due to the back-dipping low-permeability strata (opposite to the main groundwater flow lines), which act as hydraulic barriers. According to findings related to low-permeability fault zones [15], the relatively high head loss observed within the turbidite succession would be distributed across several aquitards characterized by relatively low integrity (*sensu* [49]).

Conversely, possible low-permeability normal fault cores seem not to play a significant role in (partially or totally) impeding groundwater flow (according to the absence of springs along the Figliarola tectonic discontinuity oriented sub-perpendicular to the groundwater flow lines), unlike observations made in other sedimentary aquifer systems (e.g., [14, 15]) and unlike findings related to reverse faults in turbidite systems (e.g., [8]).

The conceptual model described above also agrees with the yearly variations in spring water temperature due to groundwater flow within the shallower heterothermic zone, as well as the presence of microbial communities characterized by mainly mesophilic or psychrophilic bacterial genera. Moreover, each of the springs monitored in this study is the partial outflow of the shallower groundwater component flowing close to the ground (Fig 15).

As per the microbiological features of spring waters, according to findings in other hydrogeological settings [47], the detection of fecal bacteria demonstrates the hydraulic interconnection between the topsoil and the springs, suggesting the existence of a pore/fracture network (within the low-permeability rocks) whose size is greater than that of bacterial cells (in the order of one to several microns), and the absence of significant mechanical filtration (e.g., [51–53]). Methodologically, these findings further support the effectiveness of microbial communities as natural tracers in hydrogeological studies [54], even when studying migration in low-permeability heterogenous media (e.g., [23]).

However, (i) the coexistence of two main spring regimes (smoothed and irregular), (ii) the existence of direct and inverse relationships between spring discharge and spring water EC, and (iii) the observation of constant and variable isotopic signals in water samples collected at different springs, suggest that the aquifer system is characterized by overall low permeability, even though higher permeability portions exist locally, according to the lithological (and hydraulic) heterogeneity of the turbidite system. Moreover, at the very local scale, the soil/rock permeability can be enhanced due to (i) the effect of weathering and/or stress-release fracturing (e.g., [8, 55–57]) on bedrock portions too thin to be detectable by geophysical investigations, as well as (ii) the activity of soil arthropod community, which can increase both effective porosity and permeability in shallow low-permeability media (e.g., [58]).

The hydrogeological conceptual model described above is also in agreement with the hydrochemical features of the analyzed stream and spring waters. PCA was reported as a useful tool to describe hydrochemical data (e.g. [59–61]). According to the PCA1 plot (Fig 10) and relative PERMANOVA, samples of stream water present distinct hydrochemical characteristics compared to spring water samples, despite isotopic and hydrological results underlining a groundwater input to channel flow. Thus, the flow increase cannot be ascribed only to a diffuse groundwater contribution, but also to the precipitation runoff. In fact, at the upstream sampling point (F001), higher tritium content was measured, while the lower tritium content at the downstream sampling point (F005) underlines a more consistent groundwater contribution to channel flow. This is consistent with the study area slope, watershed area (1.18 km^2^ using F005 as outlet point), and the low bulk permeability of the studied system. In addition, these interpretations underline once again how the interdisciplinary approach can lead to a better understanding of hydrogeological system behaviors, especially when compared to those obtained from single discipline investigations. The PCA2 and consequent PERMANOVAs results underlined underlying hydrochemical differences among the groundwater samples. In fact, PC1 well described the dissimilarities between recession and recharge periods (Fig 11). Similarly, both PC1 and PC2 capture differences among spring water samples from different members of the Albidona Formation (Fig 12). Both PCAs reported marked differentiations among the investigated samples, successively confirmed by PERMANOVAs. The processes controlling this distinctness remain unexplored. In fact, the aim of these analyses was not to characterize processes affecting groundwater, but to outline differences among data from the investigated area. Although these are just preliminary and exploratory results, interesting associations between analyzed variables have been found, demanding further and more specific investigations, which will be performed to define the hydrogeological, biochemical, and geochemical processes typifying this study area. As example, a detailed geochemical conceptual model of the area will be implemented, linking the mineralogical data with the chemical characteristics of the surface- and spring water through specific analyses.

**Fig 16:**
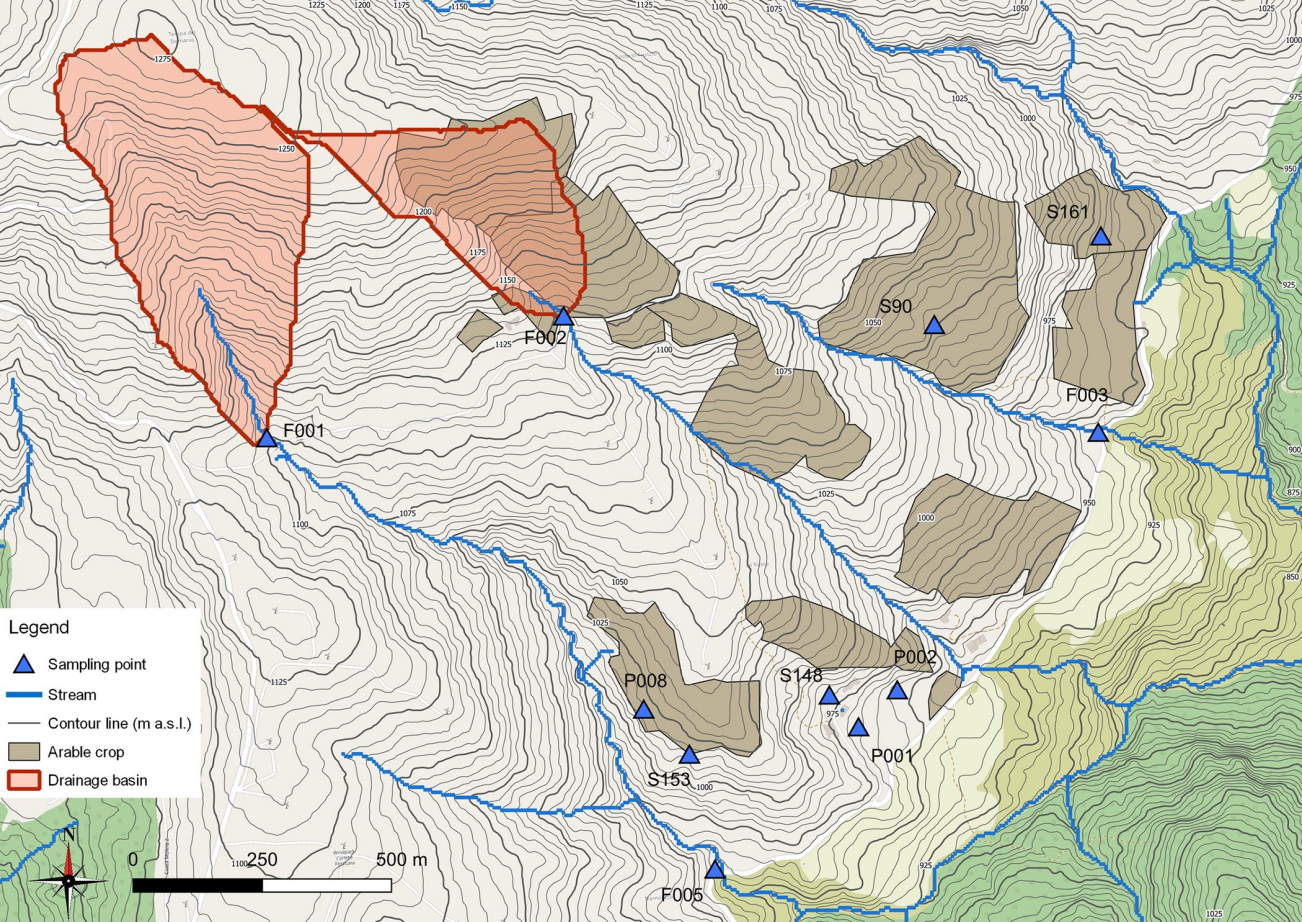
Arable crops in the study area and watersheds of F001 and F005. Base map and data from OpenStreetMap and OpenStreetMap Foundation.

In the area, several agricultural areas were identified. Thus, considering the heterogeneity of the aquifer and the low difference between groundwater and ground level, we evaluated if the fertilization performed in the area could result in NO_3_^-^ contamination of spring waters. In the area only extensive agriculture is performed, but the relatively small entities of the groundwater hosted in the turbiditic aquifer can be easily contaminated by low amount of fertilizer. Nevertheless, in all the analyzed samples (Table 3, S1 Table), NO_3_^-^ concentrations were below the limits imposed by European directives ([62, 63]). This result excludes the more likely anthropic contamination source of groundwater in the area and eutrophication issues of surface waters fed by these springs. Although most springs are near cultivated crops, their NO_3_^-^ concentrations were highly variable, providing another piece of information about the aquifer heterogeneity. Given the hydrogeological characteristics of the study area, it can be reasonably speculated that the groundwater table has a morphology similar to the topography of the investigated area. This justifies the different NO_3_^-^ concentrations between P002 and P001, while differences with S148 can be associated to the different lithological and hydraulic features of the sub-system that feeds this spring, as already noted by PCA2 (Fig 12). P008 and S153 showed similar concentrations, thus suggesting a common NO_3_^-^ source, but also lithological and mineralogical sub-system, even though the hydraulic features vary locally causing significant differences in hydraulic regime and EC variations over time. Lastly, springs S90 and S161 were characterized by lower NO_3_^-^ concentrations, underlying the absence of fertilization in the area or the high denitrification rate in groundwater, the latter associated to several bacterial genera detected in the analyzed spring water samples. Regarding surface waters, there is a clear difference in NO_3_^-^ concentrations between F001 and F002 (S1 Table). This dissimilarity can be ascribed to the presence of arable crops in the F002 watershed (Fig 16), which could cause nitrogen loss due to runoff and leaching compared to the F001 watershed, where there is no cultivated soil.

### New insights and open questions

The comparison between the results of this interdisciplinary study and those obtained by other authors in different turbidite systems further confirms that the lithologic, hydraulic, and geomorphological heterogeneity of turbidite reliefs can lead to (partially) different hydrogeological conceptual models. In more detail, this comparison inspires new questions concerning how much the hydrogeological behavior of turbiditic aquifers is linked to different depositional environments, and/or is influenced by post-depositional tectonic/geomorphic/climatic processes, as well as by bio weathering [64, 65]. To answer these questions, new purpose-designed studies are being carried out in different turbidite systems within the Italian Apennine chain.

Understanding the factors influencing the hydrogeological behavior of such aquifers is of utmost importance in forecasting how these systems will respond to changing conditions, with an emphasis on climate change in the Mediterranean region (e.g., [66, 67]). For example, in a system similar to that tested in the present study, the hydraulic head lowering expected as a consequence of a lower effective infiltration could lead to the disappearance of several springs linked to the intersection between the groundwater surface and the ground, thereby causing a negative impact from both the ecological and the human perspective: (i) several groundwater-dependent ecosystems could dry out in a relatively narrow area, leaving the surrounding biota to perish, and (ii) water supply for agriculture and livestock purposes could require drilling several low-efficiency wells in the low-permeability medium. Given the possible factors influencing the hydrogeological conceptual models of these turbidite systems, more detailed investigations are required, especially at the very local scale as in the present studyIn fact, preserving the socio-economic needs of local populations and/or restoring aquatic ecosystems will demand the creation of management guidelines for national and local administrations, allowing them to better face the future challenges consequent the above-mentioned necessities.

## Supporting information

S1 Fig Interdisciplinary approach conceptual chart. Conceptual chart with the main steps of the interdisciplinary approach proposal.
(TIF)

S2 Fig Phylum level microbial community composition in samples collected from streams and springs. (TIF)

S3 Fig Genus level microbial community composition in samples collected from streams and springs. (TIF)

S1 Appendix Chemical analysis information. (DOCX)

S1 Table NO_3_^-^ concentrations in F001 and F005. Concentrations are expressed as mg/L NO_3_^-^ and NA represents not available data.
(XLSX)
