# Supplementary figures and images for: How do turbidite systems behave from the hydrogeological point of view? New insights and open questions coming from an interdisciplinary work in southern Italy
Source: PLoS One. 2022 May 6;17(5):e0268252. doi: 10.1371/journal.pone.0268252 (PMC9075667; doi:10.1371/journal.pone.0268252)

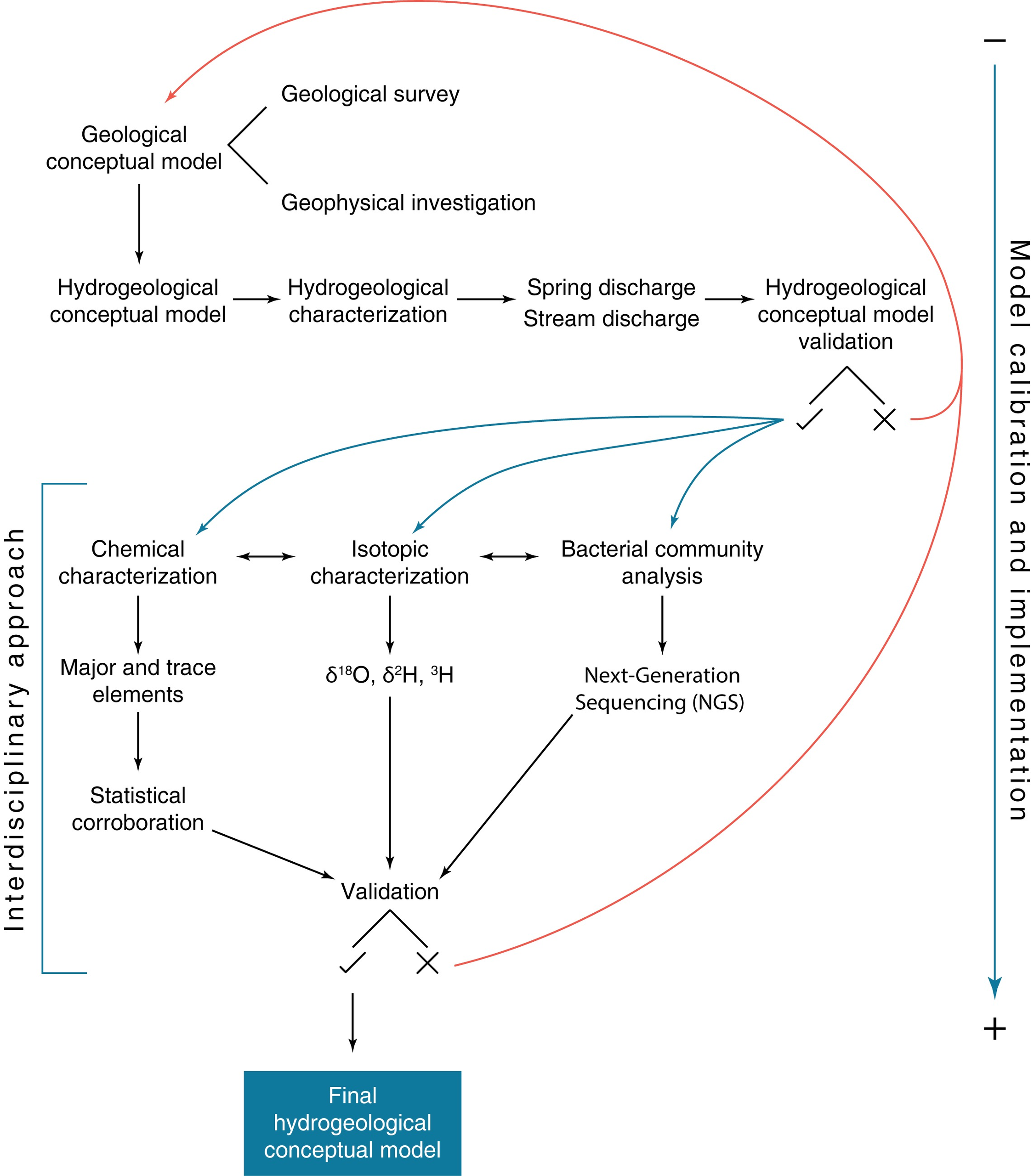

Supplement: S1 Fig — Conceptual chart with the main steps of the interdisciplinary approach proposal. (TIF) [file pone.0268252.s001.tif]

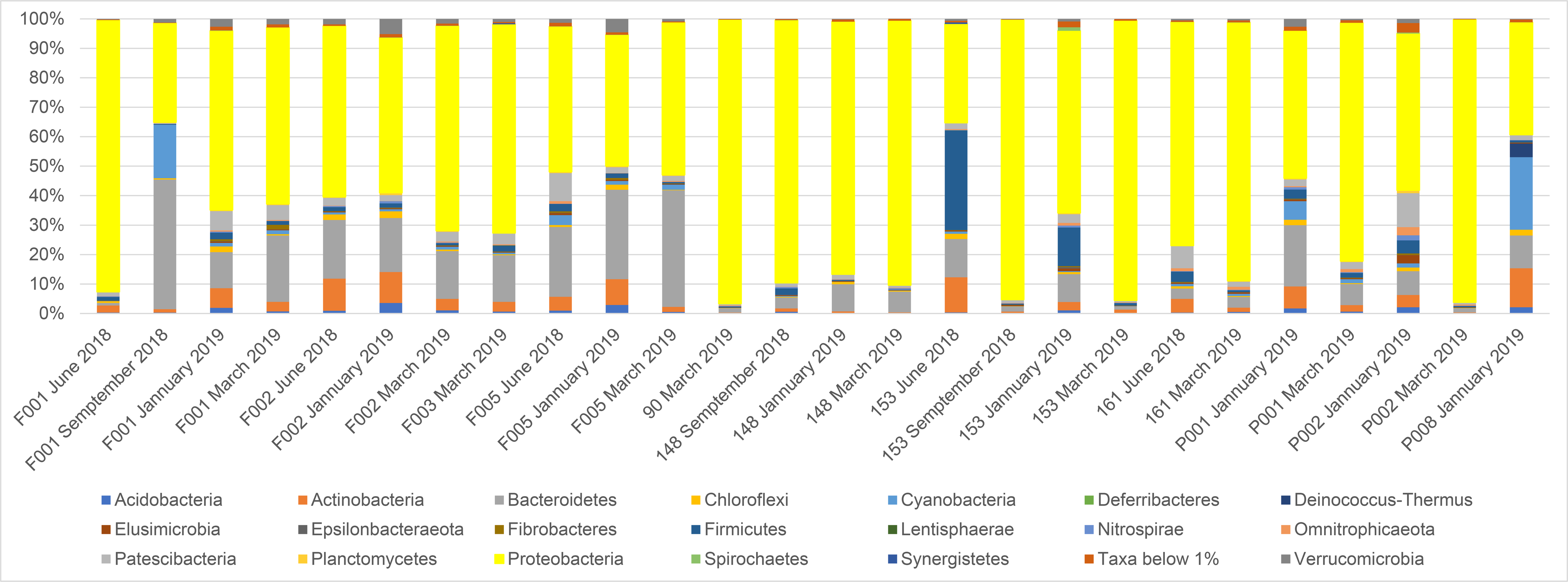

Supplement: S2 Fig — (TIF) [file pone.0268252.s002.tif]

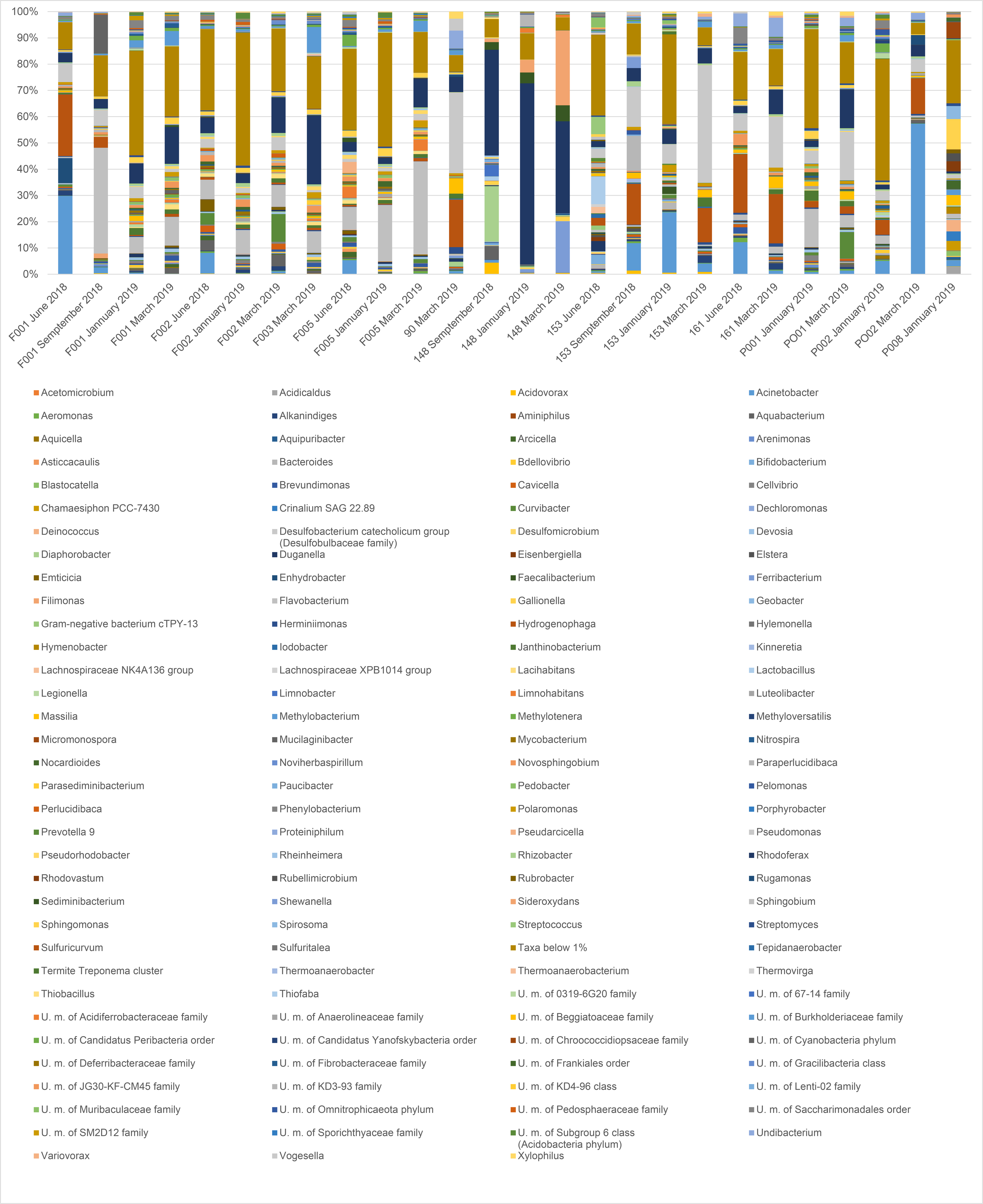

Supplement: S3 Fig — (TIF) [file pone.0268252.s003.tif]
