# Supplementary material for: How do turbidite systems behave from the hydrogeological point of view? New insights and open questions coming from an interdisciplinary work in southern Italy
Source: PLoS One. 2022 May 6;17(5):e0268252. doi: 10.1371/journal.pone.0268252 (PMC9075667; doi:10.1371/journal.pone.0268252)
Supplement: S1 Appendix — (DOCX) [file pone.0268252.s004.docx]

How do turbidite systems behave from the hydrogeological point of view? New insights and open questions coming from an interdisciplinary work in Southern Italy

**Pietro Rizzo^1^, Edoardo Severini^1,*^, Antonio Bucci^2^, Federico Bocchia^1^, Giuseppe Palladino^3^, Nicolò Riboni^1^, Anna Maria Sanangelantoni^1^, Roberto Francese^1^, Massimo Giorgi^4^, Paola Iacumin^1^, Federica Bianchi^1^, Claudio Mucchino^1^, Giacomo Prosser^3^, Domenico Mazzone^5^, Dario Avagliano^6^, Francesco Coraggio^6^, Antonella Caputi^6^,** **Fulvio Celico^1^**

**1** Department of Chemistry, Life Science and Environmental Sustainability, University of Parma, Parma, Parma, Italy

**2** Department of Biosciences and Territory, University of Molise, Pesche, Isernia, Italy

**3** Department of Sciences, University of Basilicata, Potenza, Potenza, Italy

**4** National Institute of Oceanography and Applied Geophysics–OGS, Sgonico, Trieste, Italy

**5** Proger S.p.A., San Giovanni Teatino, Chieti, Italy

**6** ENI S.p.A., Viggiano, Potenza, Italy

***** edoardo.severini@unipr.it (ES)

## Chemical Analysis

**ICP-MS analysis**

The operating parameters were as follows: RF power 1400 W; coolant gas flow 15.5 L min-1; auxiliary gas flow 0.98 L min-1; nebulizer gas flow 0.87 L min-1; nickel standard Xi cones; peak jumping data acquisition mode; dwell time 100 ms; duration time 60 s; and standard resolution. Instrument optimization was performed daily with the auto-calibration procedure to assure a response of at least 80,000 cps μg -1 L for indium and 100,000 cps μg-1 L for uranium in the high mass range. 27Al, 123Sb, 107Ag, 75As, 137Ba, 11B, 111Cd, 59Co, 52Cr, 57Fe, 7Li, 202Hg, 55Mn, 60Ni, 208Pb, 63Cu, 82Se,205Tl, and 66Zn were acquired by following the UNI-EN ISO 17294-2:2016, EPA 6020B-2014 + EPA 3015-2007 methods.

Quality control monitoring was performed by analysing a stable QC sample containing Li, Co, In, U. The QC check was performed every hour. The achievement of CV < 3% was assessed, in order to avoid instrumental calibration.

**Determination of chlorine, fluorine, nitrate, and sulfate anions**

Chlorine, fluorine, nitrate, and sulfate anions using an ion chromatograph DX-100 (Dionex Corporation, Thermo Scientific, CA, USA) equipped with an Ion Pac AG14 (4 x 50 mm) guard column (Dionex Corporation,), an Ion Pac AS14 (4 x 250 mm) anion exchange column (Dionex Corporation,), and an AERS™ 500 Electrolytically Regenerated Suppressor (Dionex Corporation).
